# An inducible co-stimulator ligand (ICOSL)-based peptide therapy for proteinuric kidney diseases targeting αvβ3 integrin

**DOI:** 10.1186/s43556-026-00594-2

**Published:** 2026-10-09

**Authors:** Alexis P. Jimenez-Uribe, Yan Xu, Yanxia Cao, Ryan Spear, Kwi Hye Koh, Adrian P. Mansini, Ariana G. Alcantar, Felix Kliewe, Nicole Endlich, Jochen Reiser, Steve Mangos, Eunsil Hahm

**Affiliations:** 1 Department of Internal Medicine, Rush University Medical Center, Chicago, IL 60612 USA; 2 Morphic Therapeutic, Waltham, MA 02451 USA; 3 Department of Dermatology, Rush University Medical Center, Chicago, IL 60612 USA; 4 Department of Anatomy and Cell Biology, University of Medicine Greifswald, Greifswald, 17487 Germany; 5 University of Texas Medical Branch, Galveston, TX 77555 USA

**Keywords:** Proteinuric kidney disease, Podocyte, αvβ3 integrin, ICOSL, Peptide therapeutics

## Abstract

Dysregulated αvβ3 integrin activation in podocytes contributes to proteinuric kidney disease by disrupting podocyte architecture and the glomerular filtration barrier. However, no treatments currently exist that therapeutically target this integrin. We previously identified inducible co-stimulator ligand (ICOSL) as an endogenous antagonist of αvβ3 integrin that protects kidney function through its arginine-glycine-aspartic acid (RGD) motif, independent of its canonical role in immunity. Leveraging the therapeutic advantages of small peptides and the renoprotective function of ICOSL, we used a structure-guided approach to design a 19-amino acid human ICOSL-derived peptide (hICOSL-19) containing the RGD motif and evaluated its therapeutic potential for proteinuric kidney disease. hICOSL-19 exhibited high-affinity binding to αvβ3 integrin, similar to full-length ICOSL, and retained its αvβ3-antagonistic activity. PEGylation of hICOSL-19 (PhICOSL-19) extended its circulating half-life while preserving its antagonistic function. In murine models of acute and chronic proteinuric kidney disease, including immune-mediated glomerulonephritis and diabetic nephropathy, PhICOSL-19 treatment significantly reduced proteinuria, limited glomerular injury, and preserved podocyte architecture. In vitro and in vivo safety assessments further showed no detectable podocyte cytotoxicity, T-cell activation, or anti-drug antibody response. These findings suggest that an ICOSL-derived peptide targeting αvβ3 integrin represents a potential therapeutic strategy for proteinuric kidney diseases associated with αvβ3 integrin activation.

**Supplementary Information:**

The online version contains supplementary material available at https://doi.org/10.1186/s43556-026-00594-2.

## Introduction

Integrins play crucial roles in maintaining the integrity of the glomerular filtration barrier, partly by regulating podocyte adhesion to the glomerular basement membrane (GBM) and by modulating foot process stability [1, 2]. Accordingly, alterations in their expression or activation patterns can lead to altered podocyte function and kidney filtration impairment. Notably, the activation of αvβ3 integrin has been identified as a hallmark of nephritis in mice [3] and is implicated in the development of proteinuria in glomerular diseases, mainly in focal segmental glomerulosclerosis (FSGS) and diabetic nephropathy (DN) [4, 5], but has also been implicated in other conditions such as Fabry nephropathy [6] and IgA nephropathy [7].

The αvβ3 integrin not only recognizes ligands that contain arginine-glycine-aspartic acid (RGD)-motifs such as vitronectin, fibronectin, and osteopontin [8, 9], but it can also be activated by other non-RGD containing ligands associated with kidney disease such as triiodothyronine (T3 hormone) [10], angiopoietin-like 3 (ANGPTL3) [11], advanced glycation end-products [12], and soluble urokinase plasminogen activator receptor (suPAR) [4, 5, 12–14]. Moreover, some early experimental evidence has shown that blocking αvβ3 integrin reduces proteinuria in DN and venom-induced glomerulonephritis models [15–17]. Despite its relevance, there are currently no clinically approved therapies targeting this integrin for kidney disease [18]. This is likely due to several confounding factors, including a high risk of immunogenicity of antibody-based treatments [19], the limited specificity of small molecule inhibitors [20], and the dose-dependent paradoxical effects observed with peptidomimetic antagonist [21–24].

Inducible co-stimulator ligand (ICOSL), also known as ICOSLG and CD275, is an endogenous co-stimulatory molecule primarily expressed by immune cells that is associated with T cell activation through its receptor ICOS [25]. Interestingly, ICOSL expression is upregulated in the kidney during crescentic glomerulonephritis [26] and LPS-induced kidney injury in mice [27]. However, contrary to what is expected from a participant in immune activation, the loss or blockade of ICOSL aggravates kidney dysfunction, suggesting a role in renoprotection [26, 28]. Recently, it was discovered that this renoprotective effect is independent of ICOSL’s immune activity and instead depends on interactions with αvβ3 integrin through ICOSL’s RGD-motif. Additionally, it was shown that ICOSL preferentially binds to the active form of αvβ3 integrin and acts as an endogenous antagonist of αvβ3 integrin [28].

By leveraging the advantages of peptide-based therapeutics [29], and the endogenous renoprotective function of ICOSL, we aimed to develop an ICOSL-based peptide targeting αvβ3 integrin for the treatment of proteinuric kidney disease. Proteinuria is a common manifestation of glomerular injury across both immune-mediated and metabolic kidney diseases, including glomerulonephritis and diabetic kidney disease [30], where podocyte dysfunction and disruption of the glomerular filtration barrier contribute to disease progression. We first confirmed that human ICOSL (hICOSL) binds αvβ3 integrin with greater affinity than other RGD-binding integrins. Guided by the hICOSL structure, we then designed a 19-amino acid RGD-containing peptide, hICOSL-19, which retained high-affinity αvβ3 binding and antagonistic activity comparable to full-length hICOSL. PEGylation of hICOSL-19 (PhICOSL-19) extended its plasma half-life while maintaining its antagonistic activity in vitro. We next evaluated peptide safety by assessing podocyte cytotoxicity and potential immunogenicity in vitro and in vivo. Finally, we tested the therapeutic efficacy of PhICOSL-19 in two complementary murine models of proteinuric kidney disease: a nephrotoxic serum (NTS)-induced glomerulonephritis model which recapitulates immune-mediated glomerular injury [31], and progressive diabetic nephropathy in BTBR ob/ob mice [32, 33]. In both models, PhICOSL-19 treatment reduced proteinuria and glomerular injury and preserved podocyte architecture.

## Results

### Human ICOSL (hICOSL) selectively binds to α_V_β_3_ integrin over other RGD-binding integrins

**Fig. 1:**
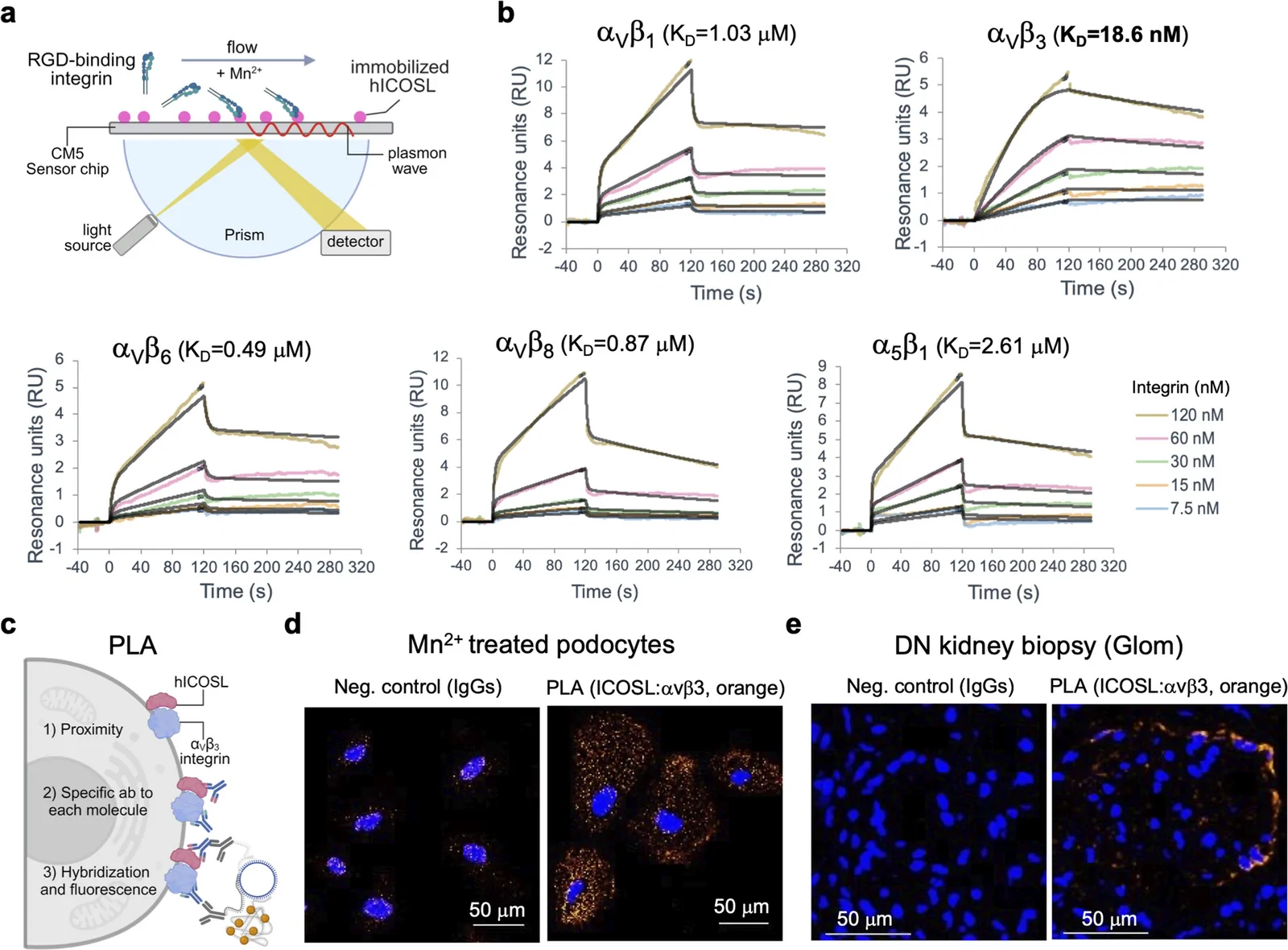
Human ICOSL preferentially binds αvβ3 integrin over other RGD-binding integrins. **a** Schematic representation of the SPR binding assay with recombinant human ICOSL (hICOSL) immobilized on a CM5 sensor chip. Different analyte proteins (RGD-binding integrins) were applied over the surface of the chip in the SPR assay buffer containing MnCl_2_ for integrin activation. **b** SPR sensorgrams showing the interactions of hICOSL with αvβ1, αvβ3, αvβ6, αvβ8, and α5β1 integrins at various concentrations (7.5, 15, 30, 60, and 120 nM) in the presence of MnCl_2_. Equilibrium dissociation constants (K_D_) were determined through kinetic fitting. **c** Schematic illustration of proximity ligation assay (PLA). **d**, **e** Representative PLA images of MnCl_2_-treated human podocytes (**d**) and a kidney biopsy from early-stage diabetic nephropathy (**e**). Orange fluorescence represents the proximity between ICOSL and αvβ3 integrin

Previously, we demonstrated that hICOSL preferentially binds to αvβ3 integrin in its MnCl_2_-induced active conformation and binds minimally when the integrin is in its inactive form [28]. To evaluate ICOSL’s binding specificity across different RGD-binding integrins, we performed surface plasmon resonance (SPR) in the presence of MnCl_2_ (Fig. 1a). hICOSL exhibited low binding affinities toward αvβ1 (K_D_ = 1.03 μM), αvβ6 (K_D_ = 0.49 μM), αvβ8 (K_D_ = 0.87 μM), and α5β1 (K_D_ = 2.61 μM) (Fig. 1b), consistent with previous reports showing no detectable binding to αIIbβ3 or αvβ5 [28]. In contrast, αvβ3 integrin bound hICOSL with high affinity (K_D_ = 18.6 nM), comparable to the binding affinity reported for the cognate ligand vitronectin (K_D_ = 8.2–12.3 nM) [34], suggesting that αvβ3 is ICOSL’s preferred integrin target.

To assess hICOSL-αvβ3 interactions at the cellular level, we performed proximity ligation assays (PLA) (Fig. 1c) in immortalized human podocytes treated with MnCl_2_. As expected, MnCl_2_-treated podocytes displayed robust orange punctate fluorescence, indicating a close proximity between hICOSL and αvβ3 integrin (Fig. 1d). Next, we used the same approach to test kidney biopsies from patients with DN. Despite a heterogeneous staining pattern, an interaction was evident throughout the kidney, with more pronounced localization within the glomerulus (Fig. 1e and Fig. S1). Collectively, these results corroborated previous findings and highlighted hICOSL’s high binding affinity and specificity for αvβ3 integrin at the molecular, cellular, and tissue levels.

### Design and functional characterization of a hICOSL-based peptide

**Fig. 2:**
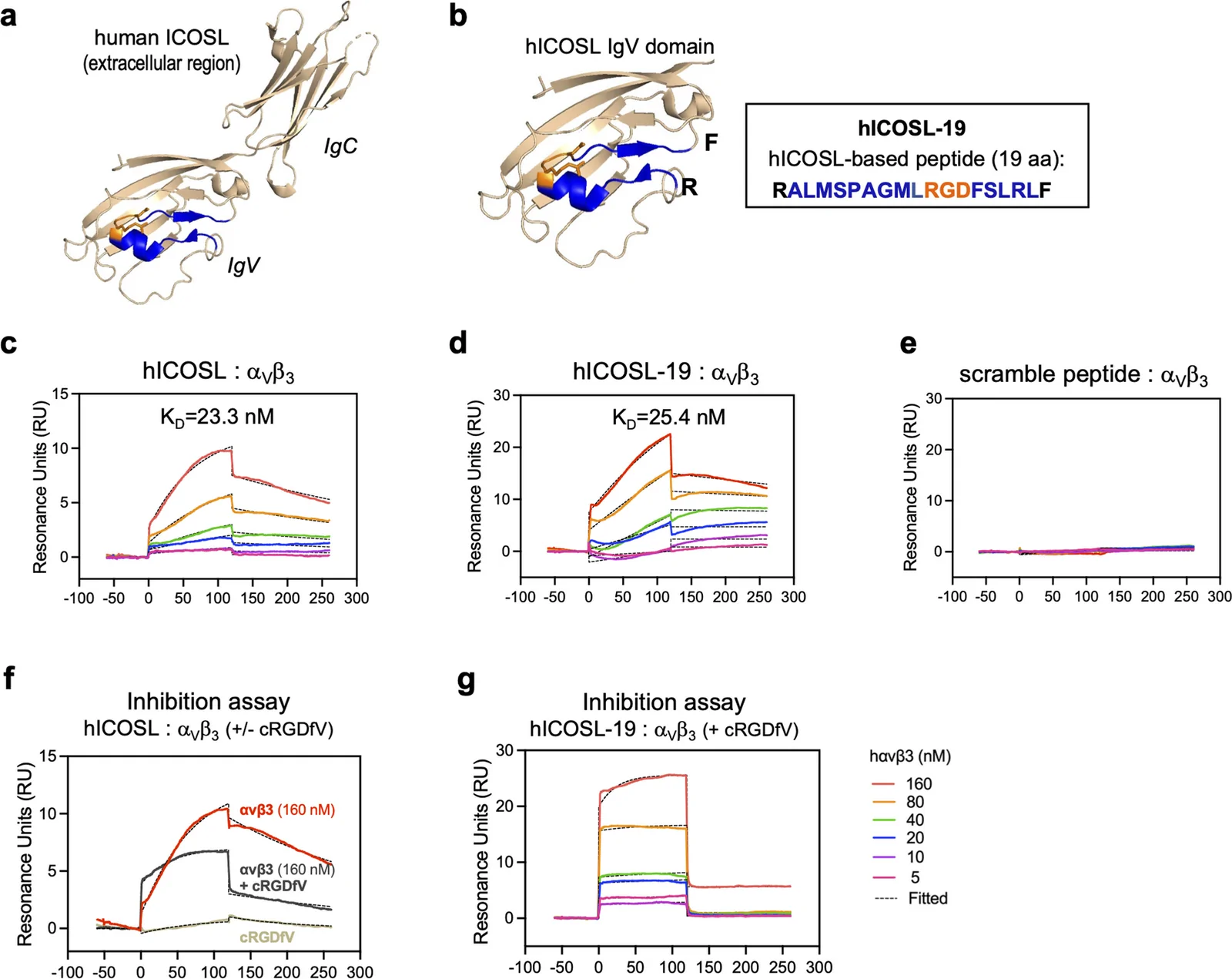
hICOSL-19 peptide binds to αvβ3 integrin and functions as an antagonist. **a**, **b** Crystal structure of hICOSL extracellular domains obtained from RCSB PDB ID 6X4G. The highlighted sequence (green) represents the 19-mer hICOSL-peptide derived from the IgV domain containing the RGD-motif (red). **c**-**e** SPR sensorgrams showing interactions of immobilized full-length hICOSL (**c**), hICOSL-19 peptide (**d**), or scrambled control peptide (**e**) with human αvβ3 integrin at various concentrations (5, 10, 20, 40, 80, and 160 nM) in the presence of MnCl_2_. The equilibrium dissociation constants (K_D_) were obtained through kinetic fitting. **f**, **g** Inhibition of αvβ3 binding to immobilized full-length hICOSL (**f**) or hICOSL-19 peptide (**g**) after pre-incubation with cRGDfV (30 μM). For **f**, human αvβ3 was applied at 160 nM; for **g**, various αvβ3 concentrations were tested

Based on our previous work and current SPR data, we sought to determine whether a hICOSL-based peptide could be developed as a therapeutic agent. Using the UniProt database [35] (entry IDs: O75144 and Q9JHJ8), we confirmed the presence of the RGD motif in both mouse (residues 237–239) and human (residues 93–95) ICOSL (Fig. S2a). Using the recently published hICOSL crystal structure [25] (PDB ID: 6X4G), we designed a 19-amino acid hICOSL-derived peptide (hICOSL-19) containing its RGD motif (Fig. 2a, b). Structural and functional mapping studies have shown that the canonical ICOS-binding interface of ICOSL is located within the membrane-distal IgV domain and involves residues clustered along the GFCC′ face, including Y51, Y53, Q55, L114, L116, and F122, as well as residues within adjacent loops [25, 36]. We therefore selected an RGD-containing region that was spatially distinct from this structurally defined ICOS-binding interface. Given that approximately 60% of peptides in clinical development are between 2 and 20 amino acids in length [37], we chose a 19-residue sequence (amino acids R83 to F101) that contained the RGD motif, had adjacent α-helical and β-sheet regions, but lacked the ICOS-binding site. The resulting hICOSL-19 peptide was estimated to have a molecular weight of approximately 2.3 kDa (Fig. 2b).

SPR analysis in the presence of MnCl_2_ showed that the hICOSL-19 peptide binds human αvβ3 integrin with high affinity (K_D_ = 25.4 nM), comparable to full-length hICOSL (K_D_ = 18.6—23.3 nM) (Figs. 1b and 2c, d). Both the full-length protein and the peptide exhibited strong binding to mouse αvβ3 integrin (K_D_ = 37.4 ± 0.4 nM and K_D_ = 44.9 ± 0.4 nM, respectively) (Fig. S2b, c). In contrast, a scrambled control peptide (also 19 amino acids long) showed no binding to either human or mouse αvβ3 integrins (Fig. 2e and Fig. S2d). The importance of the RGD-motif in mediating the interaction between hICOSL-19 and αvβ3 integrin was confirmed using an interaction inhibition assay with the cyclic pentapeptide cyclo-Arg-Gly-Asp-D-Phe-Val (cRGDfV, also known as cilengitide), a competitive blocker of RGD-binding integrins. As expected, cRGDfV effectively competed with both full-length hICOSL and the hICOSL-19 peptide for binding to αvβ3 integrin, thereby hindering the interaction (Fig. 2f, g). Steady-state affinity fitting revealed reduced binding affinity (K_D_ = 342 ± 155 nM) of hICOSL-19 to αvβ3 integrin in the presence of cRGDfV, characterized by rapid association and dissociation kinetics (Fig. 2g). Similar results were obtained with mouse αvβ_3_ integrin (Fig. S2e, f). Together, these data demonstrate that the designed hICOSL-19 peptide binds active mouse and human αvβ3 integrin with high affinity in an RGD-dependent manner, exhibiting binding kinetics comparable to full-length hICOSL while structurally lacking the key residues for ICOS-binding.

### hICOSL-19 peptide targets αvβ3 integrin on podocytes

**Fig. 3:**
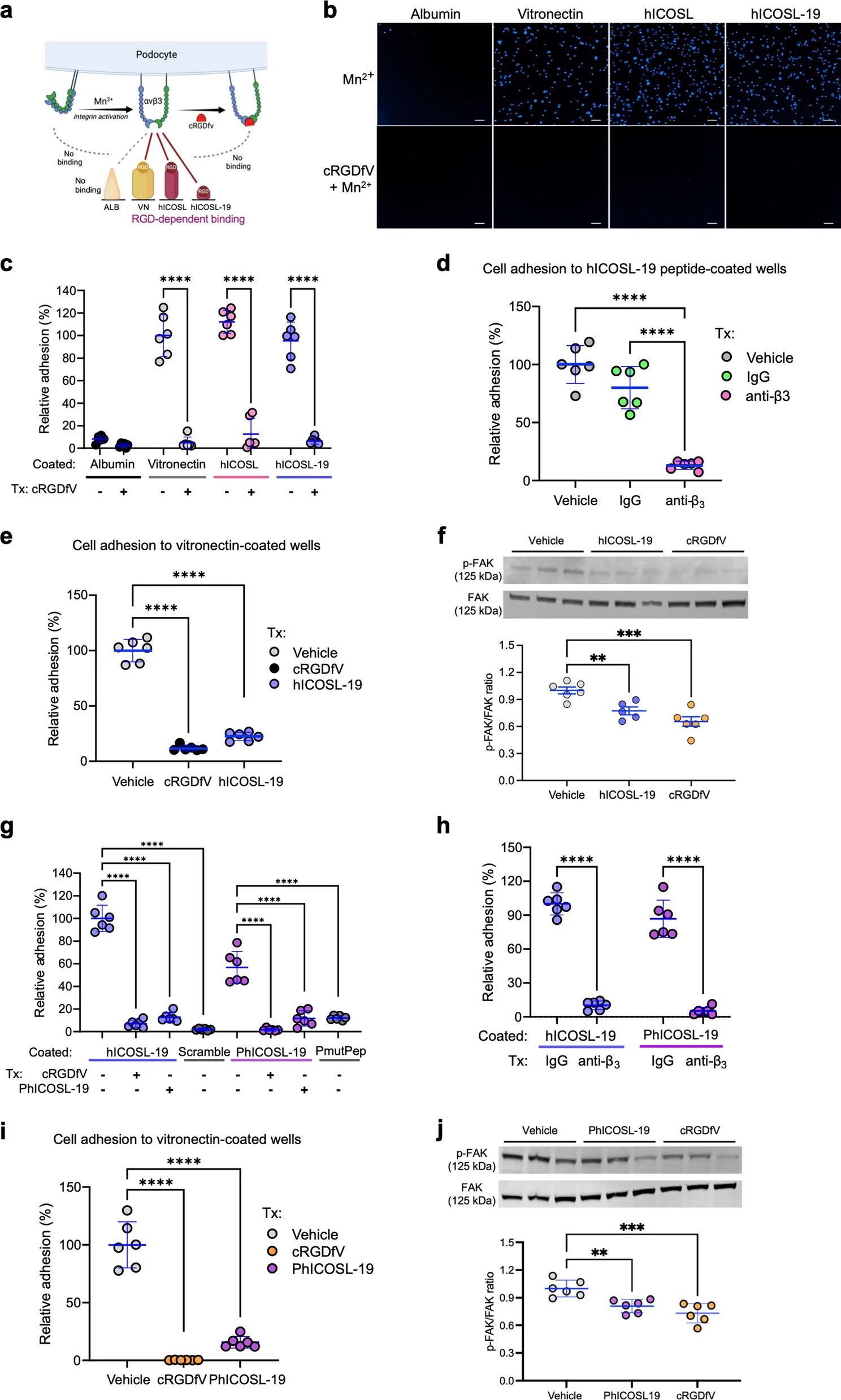
hICOSL-19 peptide targets αvβ3 integrin in podocytes, and PEGylation preserves its function. **a** Schematic of the podocyte adhesion assay. In the presence of Mn^2+^ (MnCl_2_), active αvβ3 integrin binds vitronectin (VN), full-length hICOSL, or hICOSL-19 peptide, promoting podocyte adhesion. cRGDfV or other RGD-containing molecules competitively inhibit this interaction. Albumin (ALB, no RGD motif) serves as a negative control. Illustration created with BioRender.com. **b**-**c** Representative fluorescence microscopy images (**b**) and quantification of DAPI-positive nuclei (**c**) as a measure of human podocytes adherent to plates coated with albumin (20 μg/mL, as negative control), vitronectin (1 μg/mL as positive control), full-length hICOSL (20 μg/mL), or hICOSL-19 (100 μg/mL) following Mn^2+^ stimulation (MnCl_2_ 0.25 mM), with or without cRGDfV pre-treatment (10 μg/mL). Adhesion was evaluated 2 h after seeding. Scale bar, 100 μm. **d** Relative adhesion (%) of podocytes pretreated with vehicle (PBS), IgG isotype control (10 μg/mL), or anti-β3 antibody (10 μg/mL) and seeded on hICOSL-19-coated wells. **e** Competitive adhesion assay with cells pre-treated with cRGDfV (10 μg/mL) or hICOSL-19 peptide (100 μg/mL) and seeded on vitronectin-coated wells (1 μg/mL). **f** Representative western blots and quantification of FAK phosphorylation (p-FAK/total FAK) in podocytes pre-treated with vehicle (BSA 1%), hICOSL-19 (100 μg/mL) or cRGDfV (10 μg/mL), in the presence of Mn^2+^ and seeded on vitronectin-coated plates. Cells were recovered 45 min after seeding when still in suspension, ratio normalized to vehicle pre-treated podocytes seeded on vitronectin. **g**-**h** Functional test of Un-PEGylated and PEGylated hICOSL-19 in supporting podocyte adhesion. In **g**, scrambled 19-mer control peptide and PEGylated mutant peptide (PmutPep) were coated as controls. Cells were pretreated with cRGDfV (10 μg/mL) or PhICOSL-19 (100 μg/mL). In **h**, cells were pretreated with IgG isotype control or anti-β3 antibodies (10 μg/mL). **i** Competitive adhesion assay with cells pretreated with cRGDfV or PhICOSL-19 and seeded on vitronectin-coated wells. **j** Representative western blots and quantification of FAK phosphorylation (p-FAK/total FAK) in podocytes pre-treated with vehicle or PhICOSL-19 for 20 min and seeded on vitronectin-coated wells for 45 min, normalized to vehicle. Data are mean ± SD; **d**-**g**, **i** one-way ANOVA; **c**, **h** ordinary two-way ANOVA; **j** unpaired two-tailed t-test; ****p ≤ 0.0001, **p ≤ 0.01, *p ≤ 0.05

Given that αvβ3 activation in podocytes induces structural and functional alterations [38], we investigated whether the hICOSL-19 peptide could directly target αvβ3 integrin in immortalized podocytes [39, 40] (Fig. 3a). MnCl_2_-induced activation of αvβ3 integrin promoted podocyte adhesion to surfaces coated with either full-length hICOSL or hICOSL-19 peptide, at levels comparable to the canonical αvβ3 ligand vitronectin, whereas adhesion to albumin was minimal (Fig. 3b, c). This interaction was RGD- and αvβ3-dependent, as pre-treatment with cRGDfV or an anti-β3 integrin antibody markedly reduced adhesion in both human (Fig. 3b-d) and mouse (Fig. S3a-c) podocytes. In competitive adhesion assays, pre-incubation with hICOSL-19 or cRGDfV diminished podocyte binding to wells coated with vitronectin (Fig. 3e and Fig. S3a), full-length hICOSL (Fig. S3b), or hICOSL-19 (Fig. S3c). This suggests that hICOSL-19 effectively competes with vitronectin for αvβ3 integrin binding and may participate in a competitive, potentially reversible, mechanism of integrin antagonism.

We next examined whether hICOSL-19 could antagonize αvβ3 integrin-mediated signaling by assessing focal adhesion kinase (FAK) phosphorylation, a key downstream event linked to cytoskeletal alterations and foot process effacement in podocytes [38]. As expected, pre-treatment with either cRGDfV or hICOSL-19 peptide prior to MnCl_2_/vitronectin-induced integrin activation significantly reduced FAK phosphorylation in podocytes (Fig. 3f), confirming that the hICOSL-19 peptide functionally antagonizes αvβ3 integrin signaling.

These findings prompted us to investigate the physiological role of ICOSL in the kidney, particularly under disease conditions. Building on our prior observation that ICOSL is transiently upregulated during early glomerular injury but reduced in advanced disease states [28], we examined its expression in different glomerular cell types. Inflammatory stimulation with interleukin-1 beta (IL-1β), tumor necrosis factor alpha (TNFα), or lipopolysaccharide (LPS) induced a rapid and robust increase in ICOSL expression in podocytes compared to glomerular endothelial or mesangial cells (Fig. S4a). In contrast, sustained mechanical stress [41–43] led to a marked decline in ICOSL expression in both human and mouse podocytes (Fig. S4b, c). Mining Nephroseq datasets [44] confirmed that ICOSL expression is significantly reduced in glomeruli across multiple late-stage chronic kidney diseases, including diabetic nephropathy, FSGS, IgA nephropathy, lupus nephritis, and membranous nephropathy [44, 45] (Fig. S4d). These findings suggest that early inflammation transiently boosts ICOSL as a protective response, but persistent injury drives its downregulation, supporting the idea that hICOSL-19 peptide therapy could be beneficial to restore glomerular protection.

### PEGylation enhances plasma half-life of hICOSL-19 while retaining function

A primary limitation of peptide-based therapeutics is their low bioavailability due to a short half-life. To address this, we first determined the half-life of hICOSL-19 peptide by performing plasma pharmacokinetic analysis following a single injection of fluorescein (FITC)-labeled hICOSL-19 peptide. The peptide exhibited a two-phase clearance profile (Fig. S5a), with an initial clearance (t1/2 fast = 1.3 min) followed by a terminal-phase plasma half-life (t1/2 slow = 14.96 ± 1.2 min). To improve this, hICOSL-19 peptide was modified by PEGylation at its N-terminus using 12 polyethylene glycol (PEG12) moieties, a common chemical modification to enhance bioavailability [46]. The PEGylated hICOSL-19 (PhICOSL-19) peptide, with an approximate molecular weight of 2.73 kDa, exhibited a slower initial clearance (t1/2 fast = 2.6 min) and a significantly extended terminal-phase plasma half-life (t1/2 slow = 46.74 ± 1.45 min), representing a three-fold increase in its half-life as compared to the unmodified peptide (Fig. S5b).

We next assessed whether PEGylation affected the ability of hICOSL-19 to antagonize αvβ3 integrin-mediated signaling in podocytes. Adhesion assays revealed that PhICOSL-19, like unmodified hICOSL-19, supported robust podocyte adhesion, whereas neither the scrambled control peptide nor the PEGylated RGD-mutant peptide (PmutPep; AAA substitution) promoted adhesion (Fig. 3g). Adhesion was significantly inhibited by pre-treating cells with either cRGDfV or anti-β3 integrin antibody (Fig. 3g, h, and Fig. S5c), confirming that the interaction remained RGD- and αvβ3-dependent following PEGylation. In competitive adhesion assays, pre-incubation with PhICOSL-19 peptide reduced podocyte adhesion to hICOSL-19-, PhICOSL-19-, and vitronectin-coated wells (Fig. 3g, i), indicating that PEGylation preserved αvβ3-binding capability. Consistent with these findings, PhICOSL-19 inhibited MnCl_2_/vitronectin-induced FAK phosphorylation in podocytes (Fig. 3j). Together, these results demonstrate that PEGylation enhances the pharmacologic properties of hICOSL-19 while maintaining its αvβ3 integrin antagonistic abilities.

### PhICOSL-19 peptide treatment reduces proteinuria and glomerular damage in acute and chronic kidney injury

**Fig. 4:**
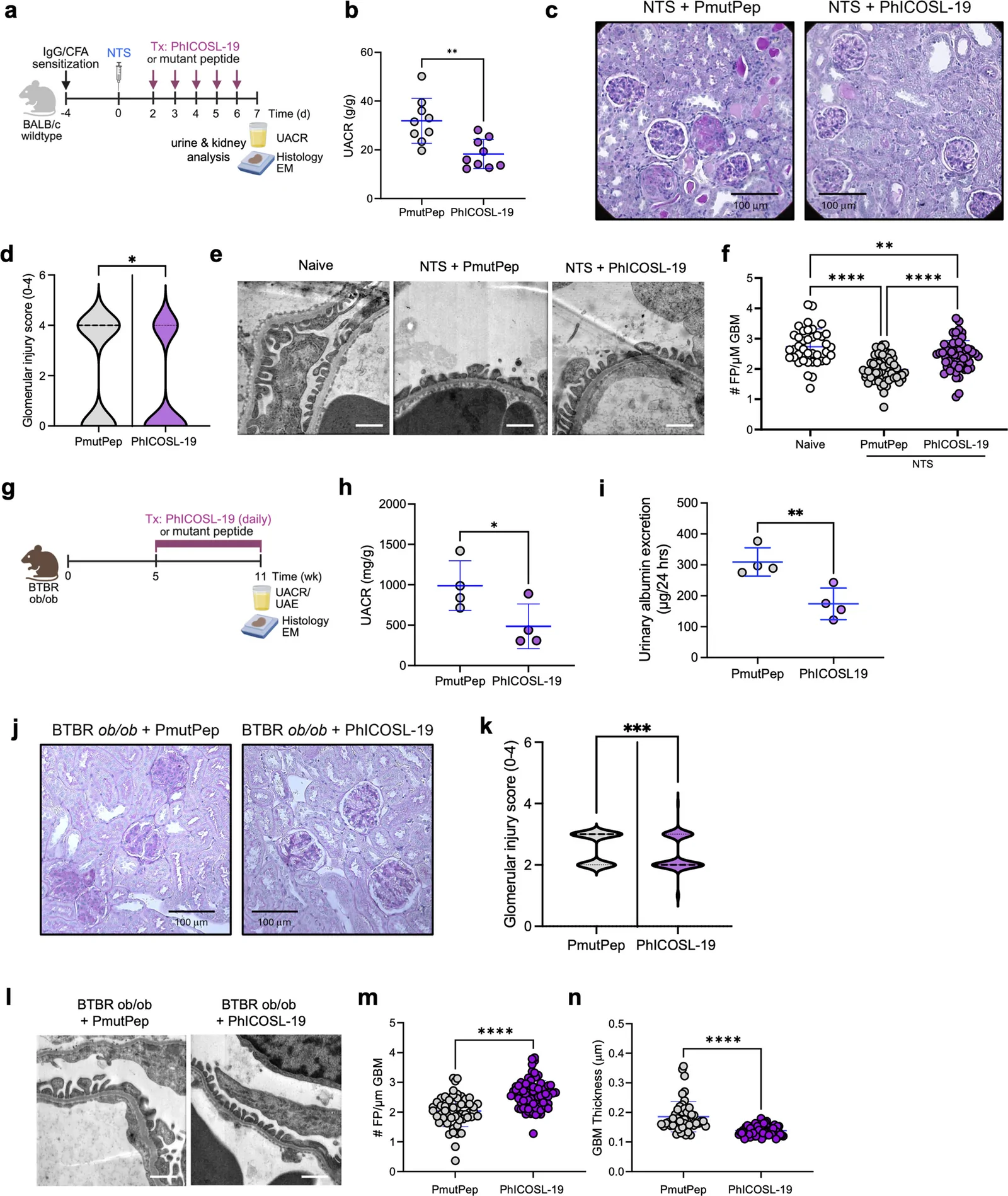
PhICOSL-19 peptide treatment reduces proteinuria and glomerular damage in chronic kidney injury models. **a** Experimental scheme of the active nephrotoxic serum (NTS) model of glomerulonephropathy. WT BALB/c mice were sensitized with sheep IgG (200 μg/mouse) in CFA 4 days before NTS injection. Two days after NTS injection, mice received daily intravenous injections of PhICOSL-19 (500 μg/kg) or PEGylated mutant peptide (PmutPep, 500 μg/kg) for 5 days. **b** Urinary albumin to creatinine ratio (ACR, *n* = 9) after treatment. Data are mean ± SD, unpaired two-tailed t-test, **p ≤ 0.01. **c** Representative PAS-stained kidney sections (40x). **d** Quantification of glomerular damage. Data are mean ± SD, unpaired two-tailed t-tests, *p ≤ 0.05. **e**, **f** Representative EM images (**e**) and quantification (**f**) of podocyte foot process (FP) effacement (#FP/μm glomerular basement membrane [GBM]) in untreated (naïve), PmutPep-treated, and PhICOSL-19-treated NTS mice. Scale bar = 1 μm. Data are mean ± SD, ordinary one-way ANOVA, **p ≤ 0.01, ****p ≤ 0.0001. **g** Experimental scheme of the diabetic nephropathy model. Five-week-old BTBR ob/ob mice received daily intravenous injections of PhICOSL-19 (500 μg/kg) or PmutPep (500 μg/kg) for 6 weeks (*n* = 4/group). **h** Urinary ACR. **i** Urinary albumin excretion (UAE). Data are mean ± SD, unpaired one-tailed t-test, *p ≤ 0.05, **p ≤ 0.01. **j** Representative PAS-stained kidney sections (40x). **k** Quantification of glomerular damage. Data are mean ± SD, unpaired two-tailed t-test, ***p ≤ 0.001. **l**-**n** Representative EM images (**l**) and quantification of FP effacement (**m**) and GBM thickness (**n**). Data are mean ± SD, unpaired two-tailed t-test, ****p ≤ 0.0001

We next evaluated the in vivo efficacy of PhICOSL-19 peptide in nephrotoxic serum [NTS; sheep anti- glomerular basement membrane (GBM) antibody]-induced glomerulonephritis. In the passive NTS model, which mimics acute glomerulonephritis, we used Icosl-KO mice that lack endogenous ICOSL expression to avoid host-related confounding effects (Fig. S6a). PhICOSL-19-treated mice showed a significant reduction in proteinuria compared with saline-treated controls after 24 and 48 h of treatment (Fig. S6b, c). To assess therapeutic potential in a more severe and persistent setting, we next employed an active NTS model that recapitulates chronic, immune-mediated kidney disease (Fig. 4a). Wildtype BALB/c mice were pre-immunized with sheep IgG and complete Freund’s adjuvant (CFA) prior to NTS injection to trigger a secondary immune activation. One day after NTS administration, mice developed severe proteinuria compared with healthy mice (Fig. S7a). The following day, proteinuric mice were treated with either PhICOSL-19 or the PEGylated RGD-mutant control peptide (PmutPep) for five consecutive days. One day after the final treatment, PhICOSL-19-treated mice showed a significant reduction in urinary albumin-to-creatinine ratio (UACR) when compared with the PmutPep group (Fig. 4b). Histological analysis demonstrated attenuated glomerular injury (Fig. 4c, d, and Fig. S7b), and ultrastructural analysis by electron microscopy (EM) revealed reduced podocyte foot process effacement, reversing the marked effacement observed in PmutPep-treated diseased mice (Fig. 4e, f).

Finally, we tested the efficacy of PhICOSL-19 peptide in a chronic, metabolically mediated model of kidney disease using BTBR ob/ob mice (Fig. 4g), which develop obesity and progressive diabetic nephropathy [32]. At 5 weeks of age, BTBR ob/ob mice showed markedly elevated UACR and 24-h urinary albumin excretion (UAE) when compared to age-matched BTBR WT controls (Fig. S7c, d), indicating significant proteinuria before treatment initiation and consistent with the original characterization of this model [32]. Daily administration of PhICOSL-19 for 6 weeks significantly reduced both UACR and UAE compared with PmutPep-treated mice (Fig. 4h, i), without affecting body weight (Fig. S7e). These functional improvements were accompanied by reduced glomerular injury (Fig. 4j, k, and Fig. S7f), decreased podocyte foot process effacement (Fig. 4l, m), and normalization of GBM thickness (Fig. 4l, n), a characteristic structural feature of diabetic nephropathy.

### PhICOSL-19 demonstrates no detectable cytotoxicity or immunogenicity

**Fig. 5:**
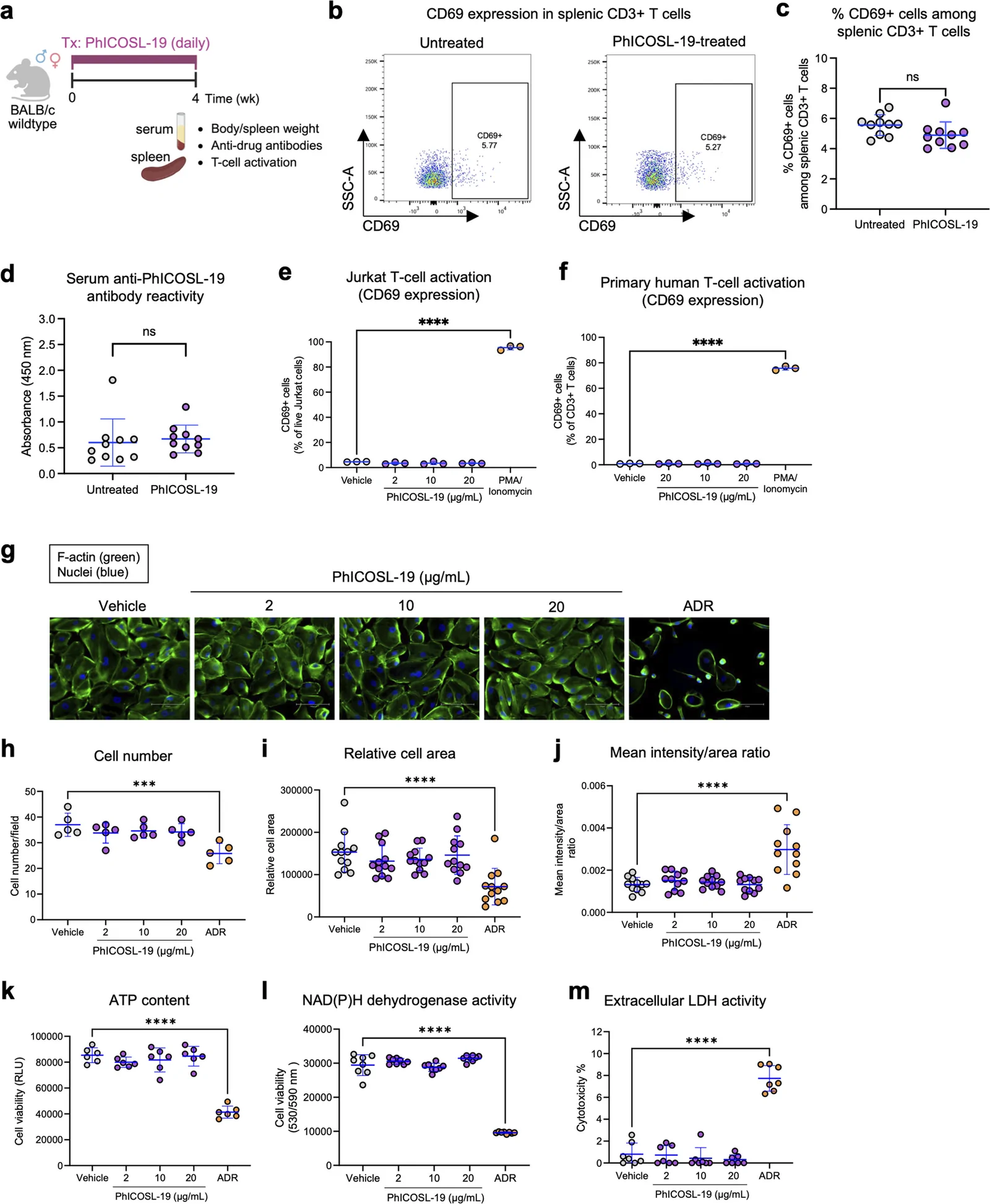
PhICOSL-19 treatment showed no detectable cytotoxicity or immunogenicity. **a** Schematic representation of the in vivo safety and immunogenicity assessment. WT BALB/c mice received daily intravenous injections of PhICOSL-19 (500 μg/kg) for 4 weeks, after which spleen and serum samples were collected for analysis. **b** Representative flow cytometry plots showing CD69 expression in splenic CD3⁺ T cells from untreated control and PhICOSL-19-treated mice. **c** Quantification of CD69⁺ cells among splenic CD3⁺ T cells. **d** Serum anti-PhICOSL-19 antibody reactivity assessed by indirect ELISA and expressed as absorbance at 450 nm. Data are presented as mean ± SD; unpaired two-tailed *t*-test. ns, not significant. **e**, **f** T-cell activation following 24-h stimulation with PhICOSL-19 (2, 10, or 20 μg/mL), PMA/ionomycin (100 ng/mL and 1 μg/mL, respectively), or vehicle (PBS). CD69⁺ cells were quantified in **e** Jurkat T cells and **f** CD3⁺ T cells from human peripheral blood mononuclear cells (PBMCs). Data are presented as mean ± SD; one-way ANOVA, ****P ≤ 0.0001. **g**-**m** Differentiated human podocytes were treated daily for 96 h with PhICOSL-19 (2, 10, or 20 μg/mL), adriamycin (ADR; 1 μg/mL), or vehicle (PBS). **g** Representative images of F-actin organization (green) and nuclei (DAPI). **h**-**j** Quantification of cell number (**h**), relative cell area (**i**), and mean fluorescence intensity-to-area ratio (**j**). **k-m** Cell viability was assessed by ATP content (**k**) and NAD(P)H-dependent dehydrogenase activity (**l**), and cytotoxicity was assessed by extracellular LDH activity (**m**). Data are presented as mean ± SD; one-way ANOVA, ****p ≤ 0.0001

To evaluate the safety and potential immunogenicity of PhICOSL-19 following repeated administration in vivo, WT BALB/c mice received PhICOSL-19 (500 μg/kg, i.v.) daily for 4 weeks (Fig. 5a). PhICOSL-19 administration did not significantly affect body weight during the treatment period (Fig. S8a), and no gross abnormalities were observed upon macroscopic organ examination. Spleen weight and length were also comparable between PhICOSL-19-treated and untreated control mice (Fig. S8b, c). Flow cytometric analysis of splenocytes showed similarly low percentages of CD69+ cells among CD3+ T cells in PhICOSL-19-treated and control mice (Fig. 5b, c), indicating no detectable increase in splenic T-cell activation. Serum antibody reactivity against PhICOSL-19 was further assessed by indirect ELISA using PhICOSL-19-coated plates. Although a modest increase in optical density was observed in serum from PhICOSL-19-treated mice, this difference was not statistically significant compared with untreated controls (Fig. 5d). Anti-PEG and anti-ICOSL antibodies were included as positive controls to confirm assay performance (Fig. S8d). Together, these findings indicate that repeated PhICOSL-19 administration was well tolerated and did not induce detectable cellular or humoral immune responses.

We next assessed the potential immunostimulatory activity of PhICOSL-19 in vitro. Jurkat T cells were exposed to different concentrations of PhICOSL-19 for 24 h, and T-cell activation was evaluated by CD69 expression, with phorbol 12-myristate 13-acetate (PMA)/ionomycin serving as a positive control. PhICOSL-19 did not significantly increase the percentage of CD69 + cells compared with vehicle treatment, whereas robust activation was observed with PMA/ionomycin (Fig. 5e). Consistent with these findings, PhICOSL-19 treatment of human peripheral blood mononuclear cells (PBMCs) did not significantly increase CD69 + T cells compared with vehicle-treated cells (Fig. 5f).

Finally, to assess potential cytotoxicity in the therapeutically relevant target cells, human podocytes were treated daily with different concentrations of PhICOSL-19 for 96 h. PhICOSL-19 did not alter F-actin organization (Fig. 5g), as reflected by no significant changes in cell number (Fig. 5h), relative cell area (Fig. 5i), or mean fluorescence intensity-to-area ratio (Fig. 5j). PhICOSL-19 also did not reduce cell viability as assessed by ATP content (Fig. 5k) and NAD(P)H-dependent dehydrogenase activity (Fig. 5l), nor did it increase extracellular lactate dehydrogenase (LDH) activity (Fig. 5m). In contrast, adriamycin (ADR), used as a positive control for podocyte injury, induced clear cytotoxic effects (Fig. 5g-j). Collectively, these in vivo and in vitro findings support a favorable preliminary safety profile for PhICOSL-19, with no detectable immunogenicity or podocyte cytotoxicity.

**Fig. 6:**
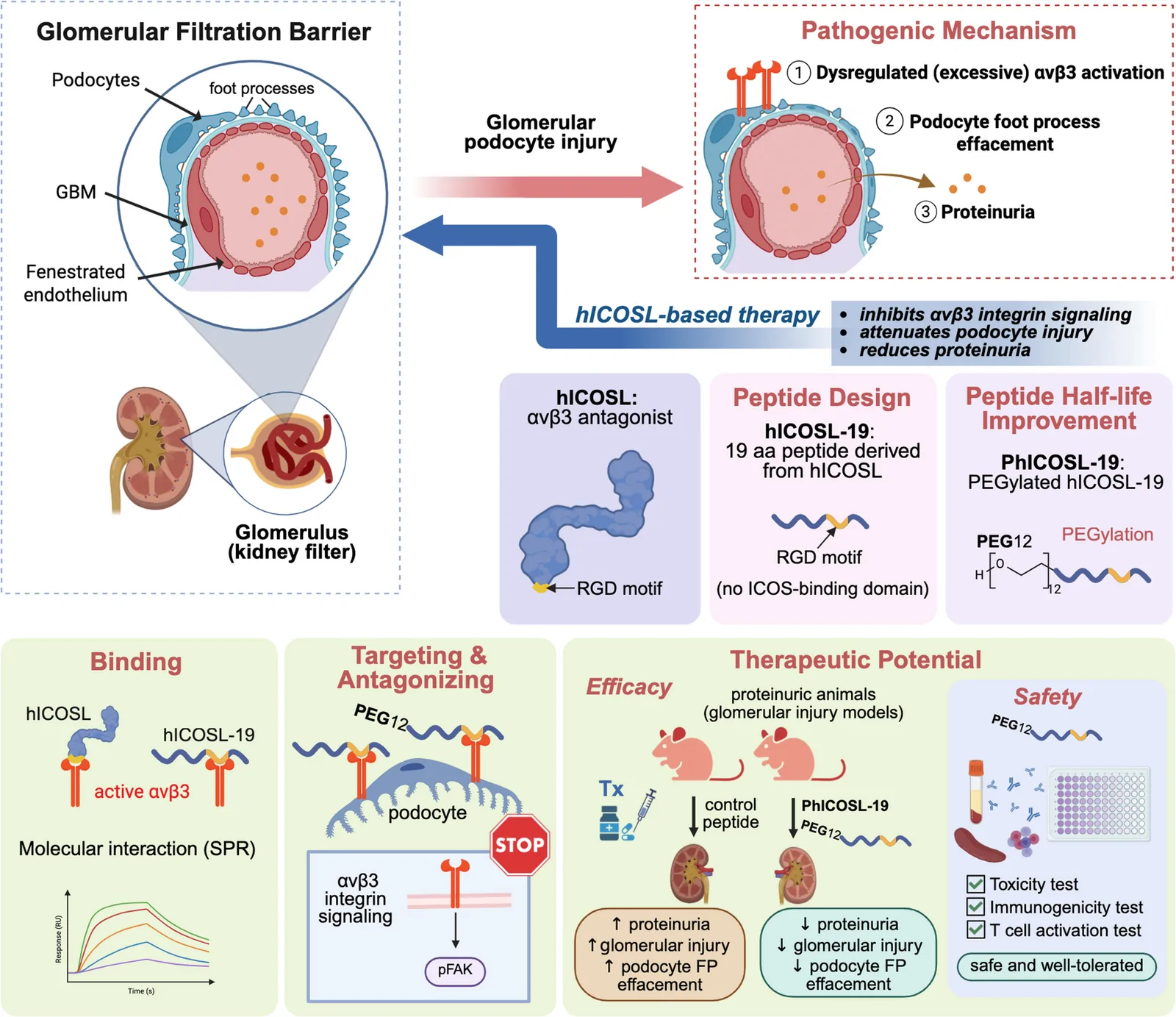
Proposed therapeutic mechanism and translational potential of the ICOSL-derived peptide targeting αvβ3 integrin in proteinuric kidney disease. Dysregulated αvβ3 integrin activation in podocytes contributes to foot process effacement, disruption of the glomerular filtration barrier, and proteinuria. ICOSL functions as an endogenous αvβ3 integrin antagonist through its RGD-containing region. Based on this mechanism, the 19-amino acid hICOSL-19 peptide was designed from the RGD-containing region of human ICOSL while excluding the ICOS-binding interface, and PEGylation generated PhICOSL-19 with an extended circulating half-life. SPR and podocyte functional assays demonstrated high-affinity αvβ3 binding and antagonism of integrin signaling, including reduced FAK phosphorylation. In murine models of proteinuric kidney disease, PhICOSL-19 treatment reduced proteinuria and glomerular injury and preserved podocyte architecture. Safety studies showed no detectable podocyte cytotoxicity, T-cell activation, or anti-drug antibody response under the experimental conditions tested. Together, these findings support PhICOSL-19 as a potential αvβ3 integrin-targeting therapeutic strategy for proteinuric kidney disease. Created with BioRender.com

Taken together, our findings demonstrate that the designed ICOSL-based peptide and its PEGylated form bind and antagonize αvβ3 integrin on podocytes. Across acute and chronic kidney disease models driven by immunologic and metabolic injury, PhICOSL-19 treatment consistently reduced proteinuria, ameliorated established glomerular pathology, and preserved podocyte architecture. Combined with its favorable preliminary safety profile, these findings support the therapeutic potential of PhICOSL-19 for treating αvβ3 integrin-mediated kidney disease (Fig. 6).

## Discussion

Proteinuric kidney diseases encompass a wide range of renal pathologies with diverse underlying etiologies, including various forms of glomerulopathy. Currently available therapies often rely on immunosuppressive agents such as glucocorticoids, mycophenolate, cyclophosphamide, and calcineurin inhibitors [47]. These treatments help reduce inflammation or the activation of immune cells that arise because of the primary insult. However, due to the complexity of their etiology, these diseases often involve additional complicating factors, including the generation of autoantibodies, complicated genetic predispositions, and the production of nephrotoxic molecules [47, 48]. Hence, successful management of these diseases is often difficult.

Activation of αvβ3 integrin has been implicated in the pathophysiology of FSGS and DN and is associated with elevated levels of circulating suPAR [49, 50], an immature myeloid cell-derived soluble factor [51] that triggers podocyte foot process effacement through αvβ3 integrin activation [12, 13, 38, 49]. Consistent with its role in podocyte injury, blockade of αvβ3 integrin in experimental models of glomerular disease ameliorates glomerular dysfunction and reduces proteinuria [4, 15, 16]. Furthermore, αvβ3 integrin activation seems to be restricted to the kidney during glomerular injury [3], making this integrin an attractive target for treating glomerular disease. However, αvβ3 integrin-targeting therapies for kidney disease currently do not exist.

Efforts to target αvβ3 integrin in other diseases have led to the development of small molecules, peptides, and antibodies. One of the most extensively studied agents was the modified pentapeptide cRGDfV, commonly known as cilengitide, which was developed as an αvβ3 integrin antagonist for cancer. Peptide-based drugs can offer advantages such as high target specificity and relatively low immunogenicity [29]. Unfortunately, cilengitide failed in a phase III clinical trial, in part because its antagonistic activity could shift toward agonism at lower concentrations [21, 52]. These observations underscore the challenge of therapeutically modulating αvβ3 integrin and highlight the need for alternative approaches that restrain pathological integrin activation without promoting unintended signaling.

Peptide-based therapeutics inspired by endogenous molecules have become an important class of pharmacological agents, with more than 40 peptides approved over the past two decades for diverse applications, including hormone analogs, growth factors, ion channel ligands, and antimicrobial agents [29]. In nephrology, however, peptide-based therapies remain relatively limited. Examples include semaglutide, a glucagon-like peptide-1 receptor agonist (GLP1-RA) [53–55], and pegmolesatide, a synthetic PEGylated erythropoietin (EPO)-mimetic peptide that activates the erythropoietin receptor for the treatment of CKD-associated anemia [56]. Beyond their established metabolic effects, GLP-1RAs have demonstrated cardiovascular and kidney benefits in patients with type 2 diabetes and CKD, including reductions in albuminuria and the risk of adverse kidney outcomes [53–55]. These examples illustrate how endogenous biological molecules and pathways can serve as templates for the development of peptide-based therapeutics with optimized pharmacological properties [57, 58].

Podocytes themselves express several molecules traditionally associated with immune or other cellular systems that can acquire distinct local regulatory functions. For example, CD80, a costimulatory molecule that typically binds to CD28 in T cells, can interact with Neph1 in podocytes and disrupt the slit diaphragm [59]. Another example is the cell surface receptor tissue factor (TF). It has been reported that TF is expressed by podocytes and interacts with the type I interferon receptor (IFNAR) to restrain its activation in a renoprotective manner [60], deviating from its canonical role as part of the extrinsic coagulation pathway. Similarly, we previously described an endogenous renoprotective role for ICOSL, where it can antagonize αvβ3 integrin activation through its RGD-motif, a function entirely independent of its canonical role in immunity [28].

Based on the importance of αvβ3 integrin in glomerular injury, the endogenous protective activity of ICOSL, and the advantages of peptides as therapeutics, we developed a peptide based on the crystal structure of human ICOSL. This short 19-amino-acid peptide retains the RGD-motif and excludes all amino acids known to be important for ICOSL’s immune activity. In the present study, hICOSL-19 bound αvβ3 integrin with an affinity comparable to that of full-length ICOSL and antagonized αvβ3 integrin at the cellular level. These findings demonstrate that the noncanonical podocyte-protective function of ICOSL can be functionally separated from the intact immune molecule and reproduced within a short peptide sequence, providing a pharmacologically tractable means of harnessing this endogenous protective mechanism.

A major challenge for peptide therapeutics is rapid degradation and short systemic half-life [57, 58]. PEGylation increased the circulating half-life of hICOSL-19 approximately three-fold, from 15 to 47 min, while preserving its biological activity. Importantly, despite the relatively short circulating half-life of PhICOSL-19, once-daily administration was sufficient to produce significant therapeutic effects in the mouse models tested. Because dosing frequency was not directly compared, however, it remains possible that more frequent administration or more sustained peptide exposure could further enhance efficacy. Future studies will therefore evaluate dosing frequency together with additional strategies to prolong peptide stability and systemic exposure, including further PEGylation, lipidation, amino acid modification, cyclization, or albumin/Fc-binding approaches [61–63].

Most importantly, the molecular and pharmacological properties of PhICOSL-19 translated into functional kidney protection in vivo. PhICOSL-19 reduced proteinuria and glomerular injury across complementary models representing acute and chronic immune-mediated injury as well as chronic metabolic kidney disease. Notably, therapeutic benefit was observed in models in which proteinuria was established before treatment initiation, supporting a therapeutic rather than solely preventive effect. The consistency of these findings across mechanistically distinct disease settings suggests that αvβ3 integrin-mediated podocyte injury may represent a common downstream pathway that can be therapeutically targeted despite differences in the initiating insult. PhICOSL-19 may therefore have potential in glomerular diseases associated with pathological αvβ3 integrin activation and elevated circulating suPAR, including FSGS and DN, as well as other conditions associated with proteinuria, such as sepsis [64] and viral infection [65]. Given the involvement of αvβ3 integrin in other pathological processes, including various forms of cancer [23, 46], the potential applications of this strategy may extend beyond kidney disease.

The preliminary safety profile of PhICOSL-19 further supports the feasibility of this therapeutic approach. PhICOSL-19 peptide did not induce detectable podocyte cytotoxicity or T-cell activation, and antibody reactivity was comparable between control and PhICOSL-19-treated animals. Antibody reactivity detected in both groups may reflect pre-existing anti-PEG antibodies [66, 67]. Because PEG-containing compounds are widely present in plastics, detergents, foods, and ointment formulations, environmental exposure may contribute to pre-existing anti-PEG reactivity, and exposure to highly PEGylated molecules has also been reported to induce antibody production [68]. Although more extensive long-term toxicology and immunogenicity studies will be required, these findings provide initial evidence that PhICOSL-19 retains its desired biological activity without detectable cytotoxic or immunostimulatory effects.

Several limitations should be considered when interpreting these findings. The present study was designed primarily to establish proof-of-concept therapeutic efficacy rather than to optimize dosing, pharmacokinetics, or formulation. Only a single dose of PhICOSL-19 was evaluated, and dose–response relationships and dosing frequency were not systematically examined. In addition, the BTBR ob/ob efficacy study was conducted with a relatively limited sample size, and confirmation of these findings in larger, adequately powered cohorts will be important. All peptide treatments were administered intravenously, and alternative routes of administration will need to be evaluated for long-term therapy. Because CKD is typically managed with combination therapy rather than a single agent, future studies should also determine whether PhICOSL-19 provides additive or complementary benefit when combined with established therapies, including angiotensin-converting enzyme (ACE) inhibitors/angiotensin II receptor blockers (ARBs), tacrolimus, sodium-glucose cotransporter 2 (SGLT2) inhibitors, GLP-1RAs, mineralocorticoid receptor antagonists (MRAs), and other clinically relevant treatments [69]. Such combinations may be particularly attractive because PhICOSL-19 directly targets αvβ3 integrin-mediated podocyte injury, whereas currently available therapies largely act through distinct immunologic, metabolic, or hemodynamic pathways. Finally, although our studies focused primarily on podocyte αvβ3 integrin, other renal cell types, as well as infiltrating immune cells, may express αvβ3 integrin during kidney injury and could also be targeted by PhICOSL-19. Further studies will be required to define the contribution of these additional cellular targets to its therapeutic effects.

In summary, this study establishes that the noncanonical αvβ3 integrin-regulatory activity of ICOSL can be isolated from the full-length immune molecule and recapitulated within a short ICOSL-derived peptide while remaining functionally distinct from canonical ICOSL immune signaling. Pharmacological optimization by PEGylation preserved this activity and enabled meaningful protection against established glomerular injury across mechanistically distinct immunologic and metabolic models, with a favorable preliminary safety profile. Together, these findings provide proof-of-concept evidence for converting an endogenous podocyte-protective mechanism into a pharmacologically tractable therapeutic strategy and establish a new approach for directly targeting αvβ3 integrin-mediated proteinuric kidney disease.

## Materials and methods

### Cell culture

Conditionally immortalized mouse and human podocytes [39, 40] were cultured and differentiated as previously described [39, 40, 70]. Podocytes were maintained at 33 °C for proliferation and differentiated at 37 °C for 10–14 days. Unless otherwise indicated, experiments were performed using differentiated podocytes. Human podocytes were cultured in RPMI-1640 containing 10% FBS, 1 X insulin-transferrin-selenium, and 1% penicillin/streptomycin. Mouse podocytes were cultured on collagen I-coated flasks in RPMI-1640 containing 10% FBS, 1% penicillin/streptomycin, and recombinant mouse IFN-γ (10 U/mL) at 33 °C, and differentiated at 37 °C in medium containing 2% FBS without IFN-γ. Human renal mesangial and glomerular endothelial cells (ScienCell) were cultured in the manufacturer-recommended media at 37 °C. Jurkat T cells were maintained in RPMI-1640 containing 10% FBS and 1% penicillin/streptomycin at 37 °C with 5% CO_2_.

### Surface plasmon resonance (SPR)

SPR studies were performed using a Biacore T200 as previously described [28]. Full-length hICOSL [28] and custom-synthesized hICOSL-19, PhICOSL-19, and scrambled control peptides (GenScript) were immobilized on CM5 sensor chips by standard amine coupling. Human or mouse integrins were perfused at concentrations of 0–160 nM in HEPES buffer containing 2 mM MnCl_2_. For competition studies, αvβ3 integrins were preincubated with cRGDfV (30 μM). Sensorgrams were double-referenced, and binding kinetics and equilibrium affinity constants were determined using a 1:1 Langmuir binding model.

### In situ proximity ligation assay (PLA)

PLA was conducted utilizing the Duolink in Situ Orange Mouse/Rabbit kit (Sigma Aldrich) according to the manufacturer’s instructions. MnCl₂-treated differentiated human podocytes and de-identified frozen kidney biopsy specimens from individuals with early diabetic nephropathy (*n* = 7) were analyzed. Biopsy specimens were provided by Dr. David Cimbaluk, Rush University Medical Center, and their retrospective use was approved by the Rush University Medical Center IRB (no. 14051401). Samples were fixed with 4% paraformaldehyde, permeabilized with 0.1% Triton X-100, blocked, and incubated overnight with rabbit anti-ICOSL [28] and mouse anti-β3 integrin antibodies (10 μg/mL each). Matched normal IgGs served as negative controls. PLA probe ligation and amplification were performed according to the manufacturer’s protocol, and signals were visualized by confocal microscopy.

### Podocyte adhesion assay

For direct adhesion assays, hICOSL-19, PhICOSL-19, full-length hICOSL, vitronectin, or BSA were used to coat 384-well plates. Differentiated human or mouse podocytes were pretreated with the indicated peptides, cRGDfV, anti-β3 antibody, control IgG, or vehicle, followed by MnCl_2_ (250 μM), and then seeded onto coated plates for 2 h at 37 °C. Nonadherent cells were removed, and adherent cells were fixed, DAPI-stained, imaged, and quantified. Competitive adhesion assays were performed on vitronectin-coated wells following pretreatment with hICOSL-19, PhICOSL-19, cRGDfV, or vehicle. Relative adhesion was expressed as a percentage of maximal adhesion to vitronectin. Detailed concentrations are provided in the Supplementary Materials.

### Western blot

Human podocytes were pretreated with vehicle, hICOSL-19 (100 μg/mL), PhICOSL-19 (100 μg/mL), or cRGDfV (10 μg/mL) for 20 min and then plated on vitronectin-coated plates in the presence of MnCl_2_ (250 μM) for 45 min. Total FAK and phosphorylated FAK (Tyr397) were detected by Western blot using specific antibodies (Cell Signaling Technology). Signals were visualized using a LI-COR Odyssey system, quantified using ImageJ, and expressed as the phospho-FAK/total FAK ratio normalized to vehicle-treated controls.

### Animals

Icosl-KO mice backcrossed onto the BALB/c background [71] were obtained from Dr. Alison Finnegan (Rush University Medical Center, Chicago, Illinois, USA). BALB/c, BTBR ob/ob, and BTBR WT mice were obtained from The Jackson Laboratory. All experiments were conducted in accordance with the NIH Guide for the Care and Use of Laboratory Animals and approved by the Rush University Institutional Animal Care and Use Committee (IACUC protocol no. 23–066). Mice were housed in the animal facility at Rush University Medical Center under specific pathogen-free (SPF) conditions, with a 12-h light/12-h dark cycle and ad libitum access to standard chow and water. Animal rooms were maintained at approximately 72 °F and 45% relative humidity. Sample sizes were chosen based on previous experience and publications in our laboratory. Animals within each experiment were age- and sex-matched, and the number of animals used in each experiment is provided in the corresponding figure legends. Animals were euthanized by carbon dioxide inhalation or isoflurane anesthesia followed by cervical dislocation, according to the approved IACUC protocol, and tissues were subsequently collected for analysis. For all in vivo efficacy studies, PhICOSL-19 was administered at 500 μg/kg body weight. This dose was selected for initial proof-of-concept studies based on our previous efficacy studies using full-length ICOSL [28] and published dosing ranges of integrin-targeting peptides in mice [72, 73].

### In vivo evaluation of PhICOSL-19 therapeutic efficacy

PhICOSL-19 efficacy was evaluated in three complementary models of proteinuric kidney disease. In the acute passive anti-GBM nephritis model, 15-week-old female *Icosl*-KO mice received nephrotoxic serum (NTS; 1 mL/kg, IV) followed by PhICOSL-19 or saline at 1 and 24 h after challenge. Proteinuria was assessed daily for 2 days. In the chronic active NTS model, 10-week-old female BALB/c mice were pre-immunized with sheep IgG in complete Freund’s adjuvant and challenged with NTS 4 days later. Urinary albumin-to-creatinine ratio (UACR) was measured before treatment to confirm proteinuria, after which mice were randomized to daily IV PhICOSL-19 or PEGylated mutant control peptide (500 μg/kg) for 5 days. Proteinuria and renal histopathology were assessed 1 day after the final treatment. In the metabolic kidney disease model, 5-week-old male BTBR ob/ob mice with established proteinuria were randomized to daily IV PhICOSL-19 or PEGylated mutant control peptide (500 μg/kg) for 6 weeks. UACR and 24-h urinary albumin excretion (UAE) were assessed after treatment, and kidneys were collected for histopathological analysis.

### Proteinuria measurements

Proteinuria was assessed using spot urine samples in the passive and active NTS models and 24-h metabolic-cage urine collections in BTBR ob/ob mice. Urinary albumin and creatinine were measured using commercial assays, and UACR was calculated as urinary albumin divided by urinary creatinine. For 24-h collections, UAE was calculated from urinary albumin concentration and total urine volume.

### Histology

Formalin-fixed, paraffin-embedded kidneys were sectioned at 5 μm and stained with hematoxylin and eosin (H&E) and periodic acid-Schiff (PAS). Glomerular injury was scored in 20–50 glomeruli per sample using a semi-quantitative scale from 0 to 4 based on hypercellularity, matrix expansion, sclerosis, and crescent formation. Histological evaluation was performed by Dr. David Cimbaluk, Director of the Renal Pathology Laboratory at Rush University Medical Center.

### Electron microscopy

Transmission electron microscopy was performed as previously described [28]. Kidney samples were fixed, post-fixed with osmium tetroxide, dehydrated, embedded in Epon resin, sectioned, and stained with uranyl acetate and lead citrate before imaging. Three mice from each peptide-treated group and two naïve control mice were analyzed, with three glomeruli examined per mouse and multiple images obtained per glomerulus.

### In vivo safety and immunogenicity

Eight- to nine-week-old WT BALB/c mice (female, *n* = 5; male, *n* = 5) received PhICOSL-19 peptide (500 μg/kg) by daily tail-vein injection for 4 weeks, while age-matched untreated WT BALB/c mice (female, *n* = 5; male, *n* = 5) served as controls. Body weight was monitored at baseline and at 2 and 4 weeks. At study completion, splenocytes were analyzed by flow cytometry for T-cell activation using CD45, CD3, and CD69, and serum was analyzed for antibodies reactive to PhICOSL-19 using an indirect ELISA. Anti-PEG and anti-ICOSL antibodies served as positive controls. Serum antibody reactivity was compared between PhICOSL-19-treated and untreated mice.

### Statistical analysis

Statistical analysis was performed utilizing Prism 10.2.1 software unless stated otherwise (GraphPad). Significance was determined with a threshold of *p* ≤ 0.05, denoted as follows: *p ≤ 0.05, **p ≤ 0.01, ***p ≤ 0.001, ****p ≤ 0.0001, and referenced accordingly in the figure legend. T-tests were employed to compare differences between two groups. Differences among multiple groups were assessed using one-way ordinary or two-way ANOVA according to each experiment. Data are presented as means ± standard deviation (SD).

## Supplementary Information

Supplementary Material 1.

## Data Availability

All data are available in the main text or the supplementary materials. Any additional information that supports the findings of this study is available from the corresponding author upon reasonable request. This study generated unique peptide reagents, which are available from the corresponding author upon reasonable request. This study also analyzed publicly available data from the Nephroseq public database (University of Michigan), including the Ju CKD Glom Dataset (199 micro-dissected glomerular samples), as well as sequence and structural information from UniProt (Entry IDs O75144 and Q9JHJ8) and the Protein Data Bank (PDB ID 6X4G).

## References

[1] Rogg M, Maier JI, Van Wymersch C, Helmstadter M, Sammarco A, Lindenmeyer M, et al. alpha-Parvin defines a specific integrin adhesome to maintain the glomerular filtration barrier. J Am Soc Nephrol. 2022;33(4):786–808. 10.1681/ASN.2021101319. PMC8970443 35260418

[2] Maier JI, Rogg M, Helmstadter M, Sammarco A, Schilling O, Sabass B, et al. EPB41L5 controls podocyte extracellular matrix assembly by adhesome-dependent force transmission. Cell Rep. 2021;34(12):108883. 10.1016/j.celrep.2021.108883. 33761352

[3] Du Y, An S, Liu L, Li L, Zhou XJ, Mason RP, et al. Serial non-invasive monitoring of renal disease following immune-mediated injury using near-infrared optical imaging. PLoS One. 2012;7(9):e43941. 10.1371/journal.pone.0043941. PMC3458852 23049742

[4] Wei C, Moller CC, Altintas MM, Li J, Schwarz K, Zacchigna S, et al. Modification of kidney barrier function by the urokinase receptor. Nat Med. 2008;14(1):55–63. 10.1038/nm1696. 18084301

[5] Wei C, El Hindi S, Li J, Fornoni A, Goes N, Sageshima J, et al. Circulating urokinase receptor as a cause of focal segmental glomerulosclerosis. Nat Med. 2011;17(8):952–60. 10.1038/nm.2411. PMC4089394 21804539

[6] Trimarchi H, Ortiz A, Sanchez-Nino MD. Lyso-Gb3 increases alphavbeta3 integrin gene expression in cultured human podocytes in Fabry nephropathy. J Clin Med. 2020;9(11). 10.3390/jcm9113659. PMC7696179 33203029

[7] Peruzzi L, Amore A, Cirina P, Trusolino L, Basso G, Ricotti E, et al. Integrin expression and IgA nephropathy: in vitro modulation by IgA with altered glycosylation and macromolecular IgA. Kidney Int. 2000;58(6):2331–40. 10.1046/j.1523-1755.2000.00417.x. 11115067

[8] Humphries JD, Byron A, Humphries MJ. Integrin ligands at a glance. J Cell Sci. 2006;119(Pt 19):3901–3. 10.1242/jcs.03098. PMC3380273 16988024

[9] Bachmann M, Schafer M, Mykuliak VV, Ripamonti M, Heiser L, Weissenbruch K et al. Induction of ligand promiscuity of alphaVbeta3 integrin by mechanical force. J Cell Sci. 2020;133(9). 10.1242/jcs.242404. 32193334

[10] Agarwal S, Koh KH, Tardi NJ, Chen C, Dande RR, WerneckdeCastro JP, et al. Deiodinase-3 is a thyrostat to regulate podocyte homeostasis. EBioMedicine. 2021;72:103617. 10.1016/j.ebiom.2021.103617. PMC8517284 34649077

[11] Liu J, Gao X, Zhai Y, Shen Q, Sun L, Feng C, et al. A novel role of angiopoietin-like-3 associated with podocyte injury. Pediatr Res. 2015;77(6):732–9. 10.1038/pr.2015.38. 25710887

[12] Kim EY, Dryer SE. RAGE and alphaVbeta3-integrin are essential for suPAR signaling in podocytes. Biochim Biophys Acta Mol Basis Dis. 2021;1867(10):166186. 10.1016/j.bbadis.2021.166186. PMC8328937 34166766

[13] Hayek SS, Koh KH, Grams ME, Wei C, Ko YA, Li J, et al. A tripartite complex of suPAR, APOL1 risk variants and alpha(v)beta(3) integrin on podocytes mediates chronic kidney disease. Nat Med. 2017;23(8):945–53. 10.1038/nm.4362. PMC6019326 28650456

[14] Wei C, Li J, Adair BD, Zhu K, Cai J, Merchant M, et al. uPAR isoform 2 forms a dimer and induces severe kidney disease in mice. J Clin Invest. 2019;129(5):1946–59. 10.1172/JCI124793. PMC6486353 30730305

[15] Zhou X, Zhang J, Haimbach R, Zhu W, Mayer-Ezell R, Garcia-Calvo M et al. An integrin antagonist (MK-0429) decreases proteinuria and renal fibrosis in the ZSF1 rat diabetic nephropathy model. Pharmacol Res Perspect. 2017;5(5). 10.1002/prp2.354. PMC5625158 28971604

[16] Maile LA, Gollahon K, Wai C, Dunbar P, Busby W, Clemmons D. Blocking alphaVbeta3 integrin ligand occupancy inhibits the progression of albuminuria in diabetic rats. J Diabetes Res. 2014;2014:421827. 10.1155/2014/421827. PMC4217341 25389530

[17] Amann K, Haas CS, Schussler J, Daniel C, Hartner A, Schocklmann HO. Beneficial effects of integrin alphavbeta3-blocking RGD peptides in early but not late phase of experimental glomerulonephritis. Nephrol Dial Transplant. 2012;27(5):1755–68. 10.1093/ndt/gfr603. 22049183

[18] Daehn IS, Duffield JS. The glomerular filtration barrier: a structural target for novel kidney therapies. Nat Rev Drug Discov. 2021;20(10):770–88. 10.1038/s41573-021-00242-0. PMC8278373 34262140

[19] Davda J, Declerck P, Hu-Lieskovan S, Hickling TP, Jacobs IA, Chou J, et al. Immunogenicity of immunomodulatory, antibody-based, oncology therapeutics. J Immunother Cancer. 2019;7(1):105. 10.1186/s40425-019-0586-0. PMC6466770 30992085

[20] Zhang J, Wang T, Saigal A, Johnson J, Morrisson J, Tabrizifard S, et al. Discovery of a new class of integrin antibodies for fibrosis. Sci Rep. 2021;11(1):2118. 10.1038/s41598-021-81253-0. PMC7822819 33483531

[21] Reynolds AR, Hart IR, Watson AR, Welti JC, Silva RG, Robinson SD, et al. Stimulation of tumor growth and angiogenesis by low concentrations of RGD-mimetic integrin inhibitors. Nat Med. 2009;15(4):392–400. 10.1038/nm.1941. 19305413

[22] Li M, Wang Y, Li M, Wu X, Setrerrahmane S, Xu H. Integrins as attractive targets for cancer therapeutics. Acta Pharm Sin B. 2021;11(9):2726–37. 10.1016/j.apsb.2021.01.004. PMC8463276 34589393

[23] Gu Y, Dong B, He X, Qiu Z, Zhang J, Zhang M, et al. The challenges and opportunities of alphavbeta3-based therapeutics in cancer: from bench to clinical trials. Pharmacol Res. 2023;189:106694. 10.1016/j.phrs.2023.106694. 36775082

[24] Nabors LB, Fink KL, Mikkelsen T, Grujicic D, Tarnawski R, Nam DH, et al. Two cilengitide regimens in combination with standard treatment for patients with newly diagnosed glioblastoma and unmethylated MGMT gene promoter: results of the open-label, controlled, randomized phase II CORE study. Neuro Oncol. 2015;17(5):708–17. 10.1093/neuonc/nou356. PMC4482861 25762461

[25] Rujas E, Cui H, Sicard T, Semesi A, Julien JP. Structural characterization of the ICOS/ICOS-L immune complex reveals high molecular mimicry by therapeutic antibodies. Nat Commun. 2020;11(1):5066. 10.1038/s41467-020-18828-4. PMC7545189 33033255

[26] Odobasic D, Kitching AR, Semple TJ, Holdsworth SR. Inducible co-stimulatory molecule ligand is protective during the induction and effector phases of crescentic glomerulonephritis. J Am Soc Nephrol. 2006;17(4):1044–53. 10.1681/ASN.2005101022. 16540559

[27] Swallow MM, Wallin JJ, Sha WC. B7h, a novel costimulatory homolog of B7.1 and B7.2, is induced by TNFalpha. Immunity. 1999;11(4):423–32. 10.1016/s1074-7613(00)80117-x. 10549624

[28] Koh KH, Cao Y, Mangos S, Tardi NJ, Dande RR, Lee HW, et al. Nonimmune cell-derived ICOS ligand functions as a renoprotective alphavbeta3 integrin-selective antagonist. J Clin Invest. 2019;129(4):1713–26. 10.1172/JCI123386. PMC6436851 30747722

[29] Wang L, Wang N, Zhang W, Cheng X, Yan Z, Shao G, et al. Therapeutic peptides: current applications and future directions. Signal Transduct Target Ther. 2022;7(1):48. 10.1038/s41392-022-00904-4. PMC8844085 35165272

[30] Petrazzuolo A, Sabiu G, Assi E, Maestroni A, Pastore I, Lunati ME, et al. Broadening horizons in mechanisms, management, and treatment of diabetic kidney disease. Pharmacol Res. 2023;190:106710. 10.1016/j.phrs.2023.106710. 36871895

[31] Song J, Xu S, Liu W, Sheng J, Zhao Y, Li S, et al. Application of the nephrotoxic serum nephritis model in glomerulonephritis research. Mol Med. 2026. 10.1186/s10020-026-01460-1. PMC13255230 41928107

[32] Hudkins KL, Pichaiwong W, Wietecha T, Kowalewska J, Banas MC, Spencer MW, et al. BTBR Ob/Ob mutant mice model progressive diabetic nephropathy. J Am Soc Nephrol. 2010;21(9):1533–42. 10.1681/ASN.2009121290. PMC3013527 20634301

[33] Keller MP, O’Connor C, Bitzer M, Schueler KL, Stapleton DS, Emfinger CH, et al. Genetic analysis of obesity-induced diabetic nephropathy in BTBR mice. Diabetes. 2024;73(2):312–7. 10.2337/db23-0444. PMC10796299 37935024

[34] Chillakuri CR, Jones C, Mardon HJ. Heparin binding domain in vitronectin is required for oligomerization and thus enhances integrin mediated cell adhesion and spreading. FEBS Lett. 2010;584(15):3287–91. 10.1016/j.febslet.2010.06.023. 20600001

[35] UniProt C. UniProt: the universal protein knowledgebase in 2025. Nucleic Acids Res. 2025;53(D1):D609–17. 10.1093/nar/gkae1010. PMC11701636 39552041

[36] Chattopadhyay K, Bhatia S, Fiser A, Almo SC, Nathenson SG. Structural basis of inducible costimulator ligand costimulatory function: determination of the cell surface oligomeric state and functional mapping of the receptor binding site of the protein. J Immunol. 2006;177(6):3920–9. 10.4049/jimmunol.177.6.3920. 16951355

[37] Lau JL, Dunn MK. Therapeutic peptides: historical perspectives, current development trends, and future directions. Bioorg Med Chem. 2018;26(10):2700–7. 10.1016/j.bmc.2017.06.052. 28720325

[38] Lin Y, Rao J, Zha XL, Xu H. Angiopoietin-like 3 induces podocyte F-actin rearrangement through integrin alpha(V)beta(3)/FAK/PI3K pathway-mediated Rac1 activation. Biomed Res Int. 2013;2013:135608. 10.1155/2013/135608. PMC3835706 24294595

[39] Saleem MA, O’Hare MJ, Reiser J, Coward RJ, Inward CD, Farren T, et al. A conditionally immortalized human podocyte cell line demonstrating nephrin and podocin expression. J Am Soc Nephrol. 2002;13(3):630–8. 10.1681/ASN.V133630. 11856766

[40] Mundel P, Reiser J, Zuniga Mejia Borja A, Pavenstadt H, Davidson GR, Kriz W, et al. Rearrangements of the cytoskeleton and cell contacts induce process formation during differentiation of conditionally immortalized mouse podocyte cell lines. Exp Cell Res. 1997;236(1):248–58. 10.1006/excr.1997.3739. 9344605

[41] Endlich N, Kress KR, Reiser J, Uttenweiler D, Kriz W, Mundel P, et al. Podocytes respond to mechanical stress in vitro. J Am Soc Nephrol. 2001;12(3):413–22. 10.1681/ASN.V123413. 11181788

[42] Greiten JK, Kliewe F, Schnarre A, Artelt N, Schroder S, Rogge H, et al. The role of filamins in mechanically stressed podocytes. FASEB J. 2021;35(5):e21560. 10.1096/fj.202001179RR. 33860543

[43] Kliewe F, Kaling S, Lotzsch H, Artelt N, Schindler M, Rogge H, et al. Fibronectin is up-regulated in podocytes by mechanical stress. FASEB J. 2019;33(12):14450–60. 10.1096/fj.201900978RR. 31675484

[44] Nephroseq. University of Michigan, Ann Arbor, MI, 2024. https://www.nephroseq.org

[45] Ju W, Greene CS, Eichinger F, Nair V, Hodgin JB, Bitzer M, et al. Defining cell-type specificity at the transcriptional level in human disease. Genome Res. 2013;23(11):1862–73. 10.1101/gr.155697.113. PMC3814886 23950145

[46] van Witteloostuijn SB, Pedersen SL, Jensen KJ. Half-life extension of biopharmaceuticals using chemical methods: alternatives to PEGylation. ChemMedChem. 2016;11(22):2474–95. 10.1002/cmdc.201600374. 27775236

[47] Lin DW, Chang CC, Hsu YC, Lin CL. New insights into the treatment of glomerular diseases: when mechanisms become vivid. Int J Mol Sci. 2022;23(7). 10.3390/ijms23073525. PMC8998908 35408886

[48] Anders HJ, Kitching AR, Leung N, Romagnani P. Glomerulonephritis: immunopathogenesis and immunotherapy. Nat Rev Immunol. 2023;23(7):453–71. 10.1038/s41577-022-00816-y. PMC9838307 36635359

[49] Kim EY, Dryer SE. Role of formyl peptide receptors and beta-arrestin-1 in suPAR signal transduction in mouse podocytes: interactions with alphaVbeta3-integrin. Cells. 2024;13(2). 10.3390/cells13020172. PMC10814688 38247863

[50] Lupusoru G, Ailincai I, Sorohan BM, Andronesi A, Achim C, Micu G, et al. Serum soluble urokinase plasminogen activator receptor as a potential biomarker of renal impairment severity in diabetic nephropathy. Diabetes Res Clin Pract. 2021;182:109116. 10.1016/j.diabres.2021.109116. 34728182

[51] Hahm E, Wei C, Fernandez I, Li J, Tardi NJ, Tracy M, et al. Bone marrow-derived immature myeloid cells are a main source of circulating suPAR contributing to proteinuric kidney disease. Nat Med. 2017;23(1):100–6. 10.1038/nm.4242. PMC5405698 27941791

[52] Mason WP. End of the road: confounding results of the CORE trial terminate the arduous journey of cilengitide for glioblastoma. Neuro Oncol. 2015;17(5):634–5. 10.1093/neuonc/nov018. PMC4482863 25681307

[53] Mima A, Kidooka S, Nakamoto T, Kido S, Gotoda H, Lee R, et al. Effects of oral semaglutide on renal function in diabetic kidney disease: a short-term clinical study. In Vivo. 2024;38(1):308–12. 10.21873/invivo.13440. PMC10756470 38148042

[54] Gitto M, Catapano F, Francone M, Mincione G, Scialo V, Pivato CA, et al. GLP-1 receptor agonists and coronary plaques regression in diabetic patients after acute coronary syndromes. Acta Diabetol. 2026;63(2):179–91. 10.1007/s00592-025-02606-z. PMC12957045 41186740

[55] Berra C, Manfrini R, Bifari F, Cipponeri E, Ghelardi R, Centofanti L, et al. Improved glycemic and weight control with dulaglutide addition in SGLT2 inhibitor treated obese type 2 diabetic patients at high cardiovascular risk in a real-world setting. The AWARE-2 study. Pharmacol Res. 2024;210:107517. 10.1016/j.phrs.2024.107517. 39613122

[56] Zhang P, Jiang Y, Xu C, Zhou L, Zheng H, Xie D, et al. Pegmolesatide for the treatment of anemia in patients undergoing dialysis: a randomized clinical trial. EClinicalMedicine. 2023;65:102273. 10.1016/j.eclinm.2023.102273. PMC10632410 37954906

[57] Zheng Z, Zong Y, Ma Y, Tian Y, Pang Y, Zhang C, et al. Glucagon-like peptide-1 receptor: mechanisms and advances in therapy. Signal Transduct Target Ther. 2024;9(1):234. 10.1038/s41392-024-01931-z. PMC11408715 39289339

[58] Zhou EJ, Lang XL, Yang MJ, Sun HY, Hao MY, Jin J, et al. Modeling and biological evaluation of pegmolesatide, a novel and potent erythropoiesis-stimulating agent. J Asian Nat Prod Res. 2024;26(11):1339–47. 10.1080/10286020.2024.2362376. 38860546

[59] Khullar B, Balyan R, Oswal N, Jain N, Sharma A, Abdin MZ, et al. Interaction of CD80 with Neph1: a potential mechanism of podocyte injury. Clin Exp Nephrol. 2018;22(3):508–16. 10.1007/s10157-017-1489-3. 29022109

[60] Manoharan J, Rana R, Kuenze G, Gupta D, Elwakiel A, Ambreen S, et al. Tissue factor binds to and inhibits interferon-alpha receptor 1 signaling. Immunity. 2024;57(1):68-85 e11. 10.1016/j.immuni.2023.11.017. 38141610

[61] Gupta V, Bhavanasi S, Quadir M, Singh K, Ghosh G, Vasamreddy K, et al. Protein PEGylation for cancer therapy: bench to bedside. J Cell Commun Signal. 2019;13(3):319–30. 10.1007/s12079-018-0492-0. PMC6732144 30499020

[62] Lee MF, Poh CL. Strategies to improve the physicochemical properties of peptide-based drugs. Pharm Res. 2023;40(3):617–32. 10.1007/s11095-023-03486-0. 36869247

[63] Heremans J, Chevillard L, Mannes M, Mangialetto J, Leroy K, White JF, et al. Impact of doubling peptide length on in vivo hydrogel stability and sustained drug release. J Control Release. 2022;350:514–24. 10.1016/j.jconrel.2022.08.027. 35998769

[64] Nusshag C, Wei C, Hahm E, Hayek SS, Li J, Samelko B et al. suPAR links a dysregulated immune response to tissue inflammation and sepsis-induced acute kidney injury. JCI Insight. 2023;8(7). 10.1172/jci.insight.165740. PMC10132159 37036003

[65] Wei C, Datta PK, Siegerist F, Li J, Yashwanth S, Koh KH, et al. SuPAR mediates viral response proteinuria by rapidly changing podocyte function. Nat Commun. 2023;14(1):4414. 10.1038/s41467-023-40165-5. PMC10362037 37479685

[66] Chen BM, Su YC, Chang CJ, Burnouf PA, Chuang KH, Chen CH, et al. Measurement of pre-existing IgG and IgM antibodies against polyethylene glycol in healthy individuals. Anal Chem. 2016;88(21):10661–6. 10.1021/acs.analchem.6b03109. 27726379

[67] Kubesch K, Lubich C, Mascher D, Tangada S, Turecek PL. Evaluation of plasma polyethylene glycol (PEG) levels in a healthy adult population. Int J Toxicol. 2025;44(6):451–7. 10.1177/10915818251371966. PMC12589669 40916574

[68] Ichihara M, Shimizu T, Imoto A, Hashiguchi Y, Uehara Y, Ishida T, et al. Anti-PEG IgM response against PEGylated liposomes in mice and rats. Pharmaceutics. 2010;3(1):1–11. 10.3390/pharmaceutics3010001. PMC3857034 24310423

[69] Neuen BL, Heerspink HJL, Vart P, Claggett BL, Fletcher RA, Arnott C, et al. Estimated lifetime cardiovascular, kidney, and mortality benefits of combination treatment with SGLT2 inhibitors, GLP-1 receptor agonists, and nonsteroidal MRA compared with conventional care in patients with type 2 diabetes and albuminuria. Circulation. 2024;149(6):450–62. 10.1161/CIRCULATIONAHA.123.067584. 37952217

[70] Spear R, Jimenez-Uribe AP, Cao Y, Mangos S, Alcantar AG, Kim BH, et al. Dysregulated bone marrow contributes to glomerular injury through soluble factors. J Am Soc Nephrol. 2026;37(2):257–71. 10.1681/ASN.0000000828. PMC12889954 40779328

[71] Hamel KM, Cao Y, Olalekan SA, Finnegan A. B cell-specific expression of inducible costimulator ligand is necessary for the induction of arthritis in mice. Arthritis Rheumatol. 2014;66(1):60–7. 10.1002/art.38207. 24449576

[72] Khalili P, Arakelian A, Chen G, Plunkett ML, Beck I, Parry GC, et al. A non-RGD-based integrin binding peptide (ATN-161) blocks breast cancer growth and metastasis in vivo. Mol Cancer Ther. 2006;5(9):2271–80. 10.1158/1535-7163.MCT-06-0100. 16985061

[73] Yacobovich S, Tuchinsky L, Kirby M, Kardash T, Agranyoni O, Nesher E, et al. Novel synthetic cyclic integrin alphavbeta3 binding peptide ALOS4: antitumor activity in mouse melanoma models. Oncotarget. 2016;7(39):63549–60. 10.18632/oncotarget.11363. PMC5325384 27556860

