# Supplementary material for: An inducible co-stimulator ligand (ICOSL)-based peptide therapy for proteinuric kidney diseases targeting αvβ3 integrin
Source: Mol Biomed. 2026 Oct 9;7:200. doi: 10.1186/s43556-026-00594-2 (PMC13653019; doi:10.1186/s43556-026-00594-2)
Supplement: Supplementary file 1 [file 43556_2026_594_MOESM1_ESM.pdf]

An inducible co-stimulator ligand (ICOSL)-based peptide therapy for proteinuric kidney diseases targeting αvβ3 integrin

Alexis P. Jimenez-Uribe[1†], Yan Xu[1†], Yanxia Cao[1], Ryan Spear[1], Kwi Hye Koh[2], Adrian P. Mansini[3], Ariana G. Alcantar[1], Felix Kliewe[4], Nicole Endlich[4], Jochen Reiser[5], Steve Mangos[1] and Eunsil Hahm[1*]

[1]Department of Internal Medicine, Rush University Medical Center, Chicago, IL, 60612, United States

[2]Morphic Therapeutic, Waltham, MA, 02451, United States

[3]Department of Dermatology, Rush University Medical Center, Chicago, IL, 60612, United States

[4]Department of Anatomy and Cell Biology, University of Medicine Greifswald, Greifswald, 17487, Germany.

[5]University of Texas Medical Branch, Galveston, TX, 77555, United States

†These authors contributed equally.

*Correspondence: Eunsil_hahm@rush.edu

Supplementary Figures

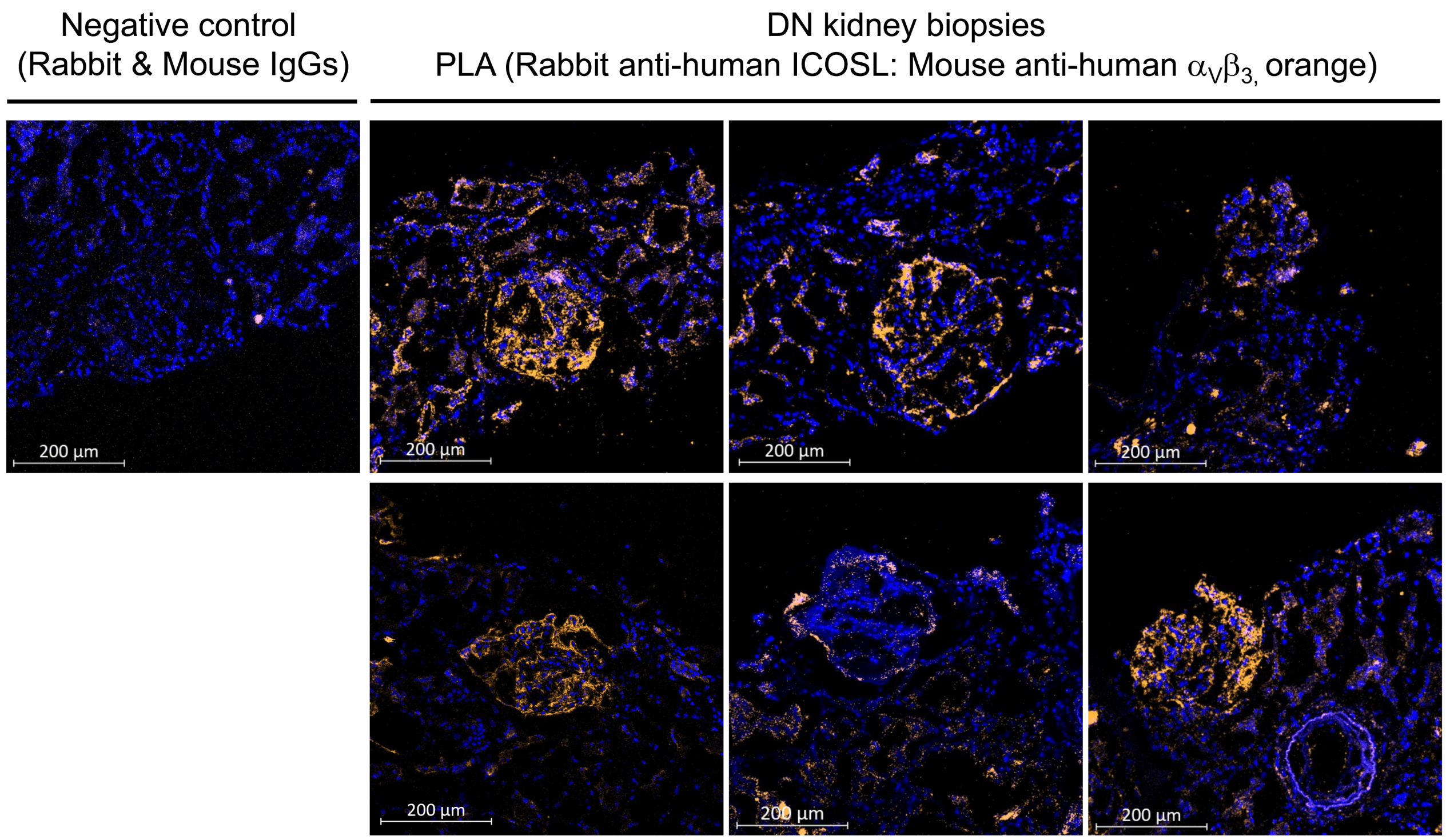

Figure S1. Interaction between hICOSL and αvβ$_3$ integrin in kidney biopsies from patients with diabetic nephropathy. Proximity ligation assay (PLA) was performed on kidney biopsies from patients diagnosed with diabetic nephropathy (n = 6). All samples were de-identified. Orange signals represent the proximity between ICOSL and αvβ$_3$ integrin.

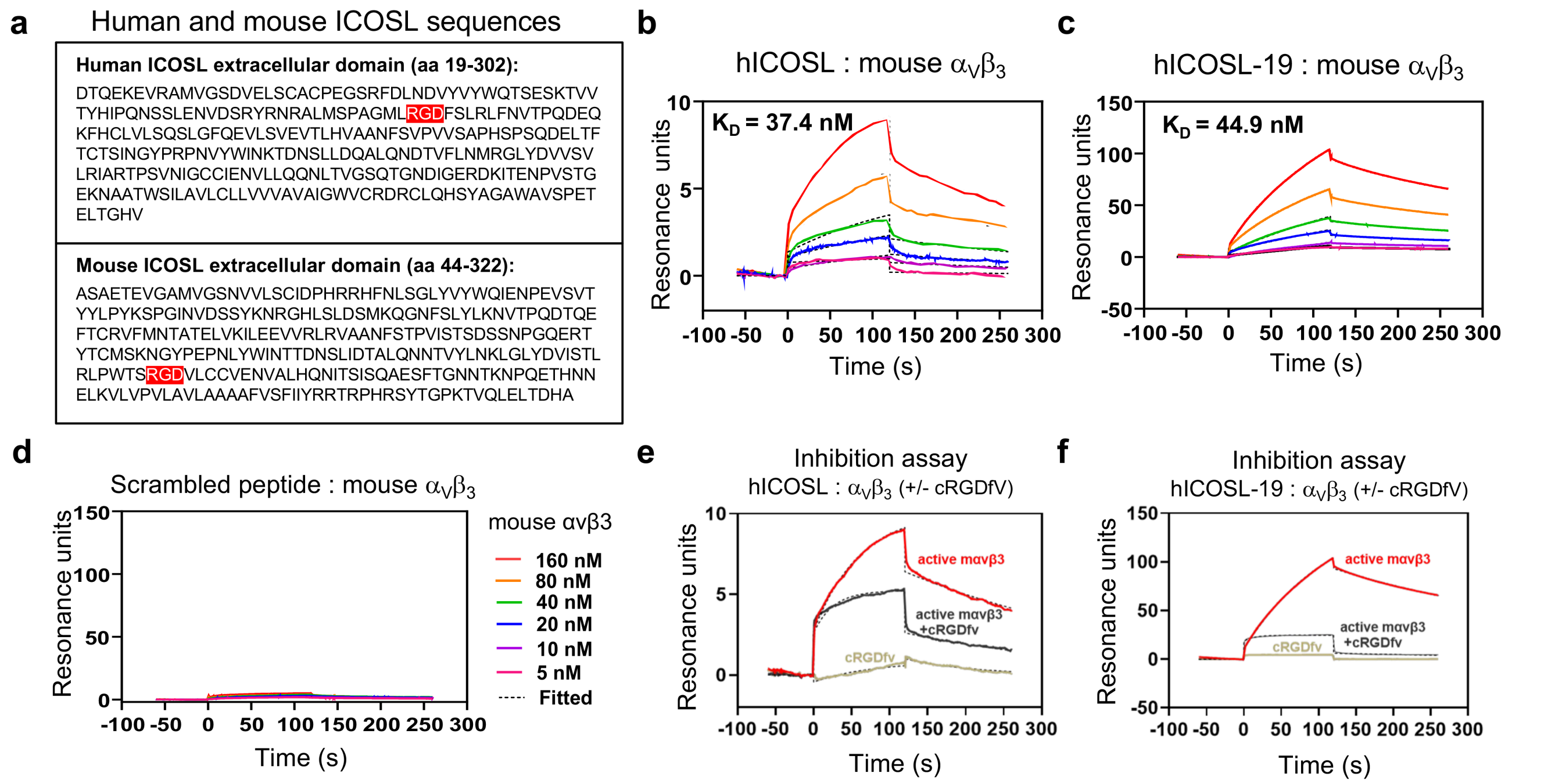

Figure S2. hICOSL-19 strongly binds to mouse αvβ3 integrin. (**a**) Amino acid sequences of human ICOSL (UniProt O75144) and mouse ICOSL (UniProt Q9JHJ8) extracellular domains highlighting the RGD-motif in red. (**b-f**) Representative SPR sensorgrams and K_D values showing interactions between mouse αvβ3 integrin and full-length hICOSL (**b**), hICOSL-19 peptide (**c**), or scrambled control peptide (**d**), in the presence of $MnCl_2$ as source of Mn^{2+} ions. Competitive inhibition assays were performed by pre-incubating mouse αvβ3 integrin (160 nM) with cRGDfV (30 μM) in the presence of $MnCl_2$, followed by perfusion through CM5 chips immobilized with either full-length hICOSL (**e**) or hICOSL-19 peptide (**f**).

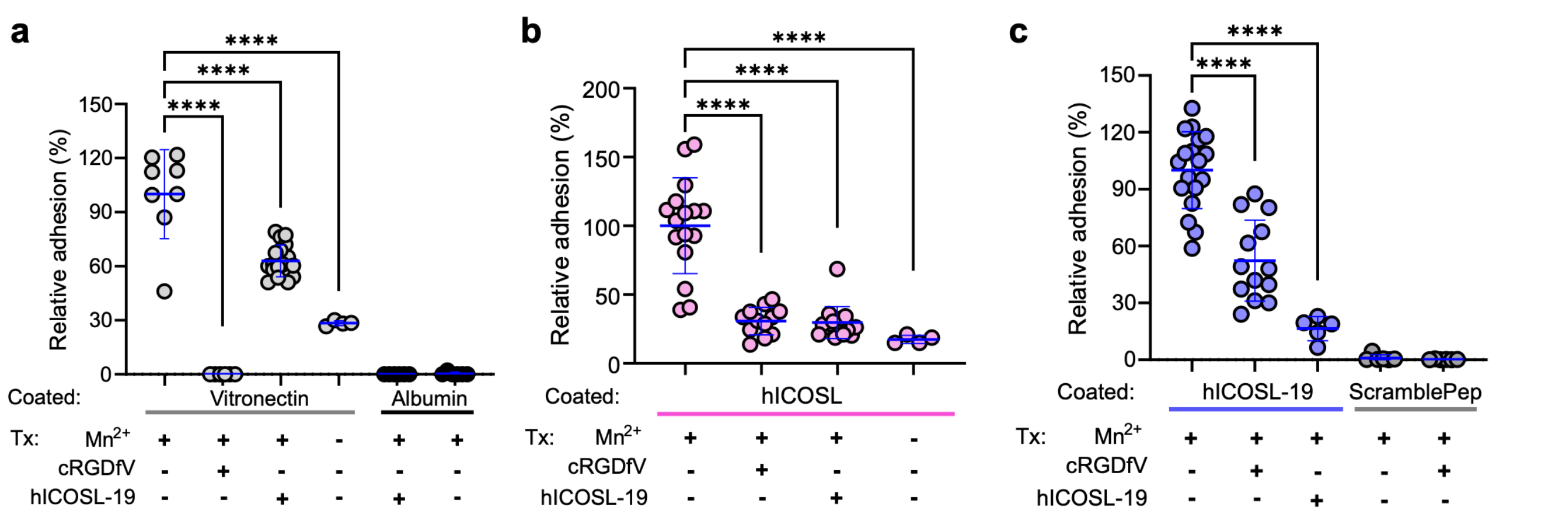

Figure S3. hICOSL-19 peptide mediates mouse podocyte adhesion in an RGD-dependent manner. Relative adhesion of mouse podocytes to wells coated with (**a**) vitronectin or albumin (negative control), (**b**) full-length hICOSL, and (**c**) hICOSL-19 or scrambled control peptides. Cells were pre-treated with PBS (vehicle) or with cRGDfV or hICOSL-19 to block RGD-dependent adhesion, in the presence or absence of $MnCl_2$ to activate integrins, as indicated. Maximum cell adhesion (average) was set as 100%, and relative adhesion in each condition was calculated accordingly. Data are shown as mean ± SD, ordinary one-way ANOVA. ***** p≤ 0.0001.*

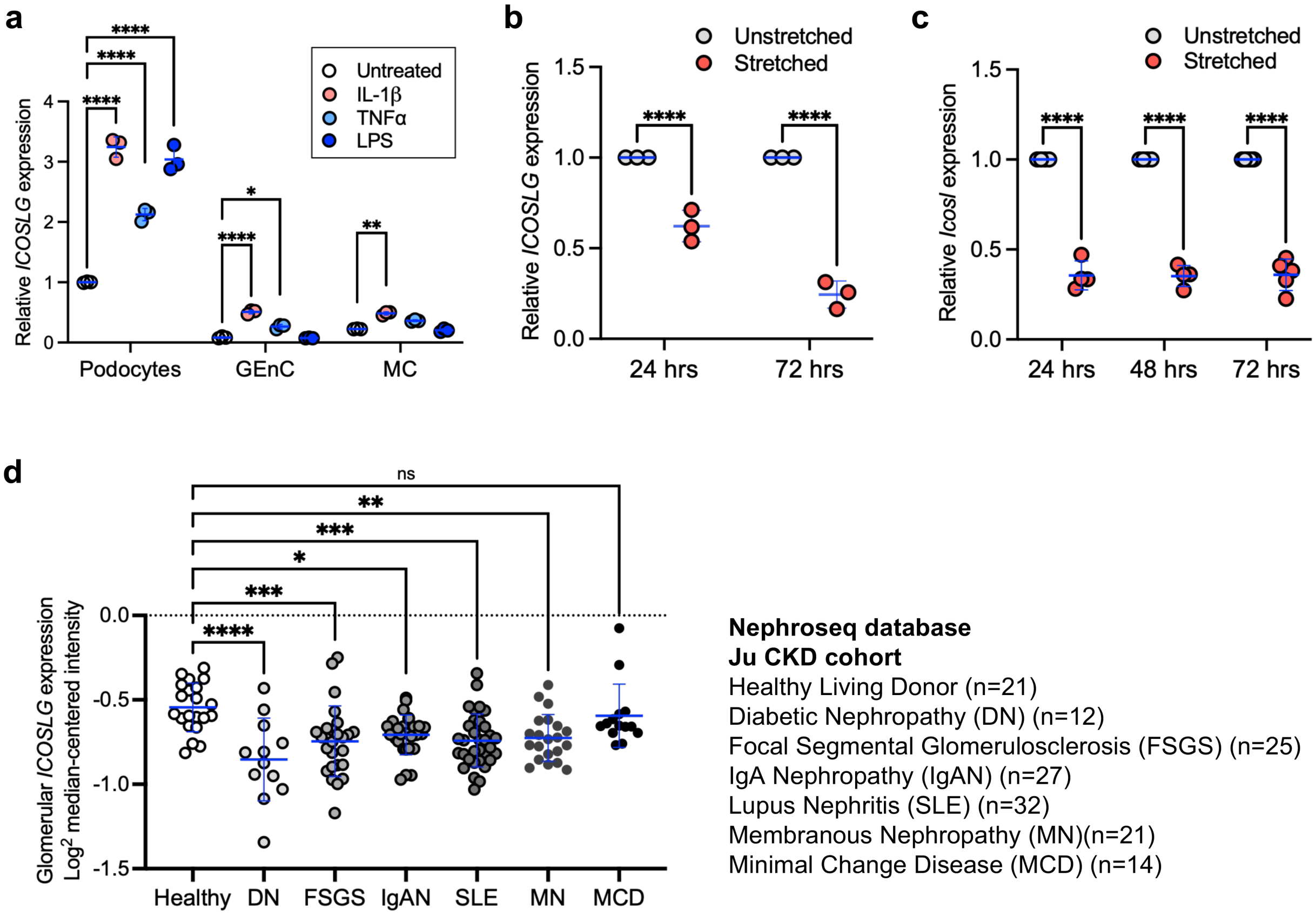

Nephroseq (The Regents of The University of Michigan, Ann Arbor, MI) was used for analysis and visualization. For further information, refer to the terms of use.
http://www.nephroseq.org/resource/main.html#ac:1N156636797.75,1N156636797.95,1N156636797.90,1N156636793.95,1N156636793.90;cv:detail;d:1N156636782;dso:geneOverex;dt:outlier;ec:[1N2,1N3];epv:1N1.1N3,1N36.1N37,1N41;et:over;f:195764547;g:23308;gt:boxplot;p:1N200015073;pg:1;pv:90;pvf:15185[ICOSL],1N800101749;scr:datasets;ss:all;v:17

Figure S4. Changes in *ICOSLG*/*Icosl* mRNA expression in glomeruli and glomerular cells. (**a**-**c**) mRNA expression was measured by quantitative PCR (qPCR) and normalized to the corresponding reference gene. (**a**) Human *ICOSLG* expression in differentiated immortalized human podocytes, human glomerular endothelial cells (GEnC), and human mesangial cells (MC) following stimulation with IL-1β (20 ng/ml), TNFα (100 ng/ml), or LPS (50 µg/ml) for 3 hours. (**b**) Human *ICOSLG* expression in differentiated immortalized human podocytes subjected to persistent low mechanical stretch (frequency 0.5 Hz), normalized to *GAPDH*. (**c**) Mouse *Icosl* expression in differentiated immortalized mouse podocytes subjected to persistent low mechanical stretch (frequency 0.5 Hz), normalized to *Gapdh*. Data are presented as mean ± SD and analyzed by two-way ANOVA. (**d**) Human glomerular *ICOSLG* expression in micro-dissected glomerular samples from patients with chronic kidney disease (CKD) of various etiologies and healthy donors. All samples were reported to be de-identified. Data were obtained from the public database Nephroseq using the Ju CKD Glom dataset. Data are presented as mean ± SD and analyzed by one-way ANOVA. *P ≤ 0.05, **P ≤ 0.01, ***P ≤ 0.001, ****P ≤ 0.0001; ns, not significant.

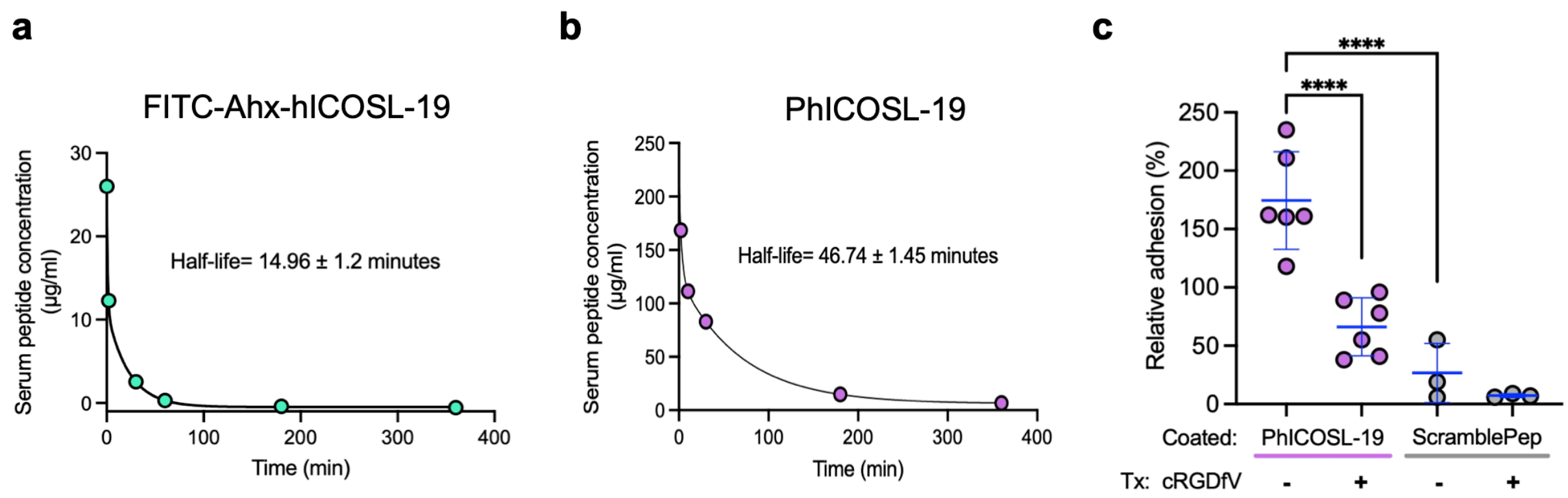

Figure S5. Pharmacokinetics of hICOSL-19 peptide and adhesion of mouse podocyte. (a-b) Pharmacokinetic analysis of FITC-conjugated hICOSL-19 (**a**) and PEGylated hICOSL-19 (PhICOSL-19) (**b**) following intravenous administration in *Icosl*-KO mice. Serum peptide concentrations were assessed by FITC fluorescence (**a**) or PEG-specific ELISA (**b**), and half-lives were determined by fitting the data to a two-phase decay model using GraphPad Prism. (**c**) Relative adhesion of mouse podocytes to wells coated with PhICOSL-19 or scrambled control peptide, as indicated. Where specified, cells were pre-treated with cRGDfV to block RGD-dependent adhesion. Relative adhesion was calculated by setting the mean maximum adhesion (average) to 100%. Data are shown as mean ± SD, ordinary one-way ANOVA, **** p≤ 0.0001.

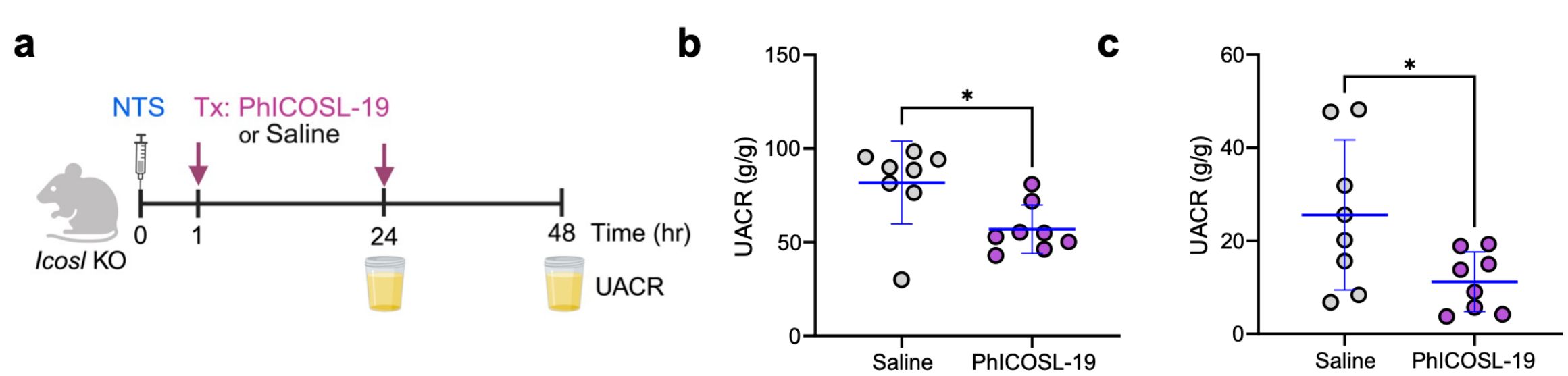

Figure S6. PhICOSL-19 treatment reduces proteinuria in a passive NTS model of acute glomerulonephritis. (**a**) *Icosl* KO mice were challenged with nephrotoxic serum (NTS, 1 mL/kg body weight) and treated intravenously with PhICOSL-19 (500 μg/kg) or saline at 1 and 24 hours after NTS administration. Urine samples were collected at 24 hours (**b**) and 48 hours (**c**) to measure urinary albumin-to-creatinine ratio (UACR) (n = 8 per group from two independent experiments). Data are shown as mean ± SD, unpaired two-tailed t-tests. *P ≤ 0.05.

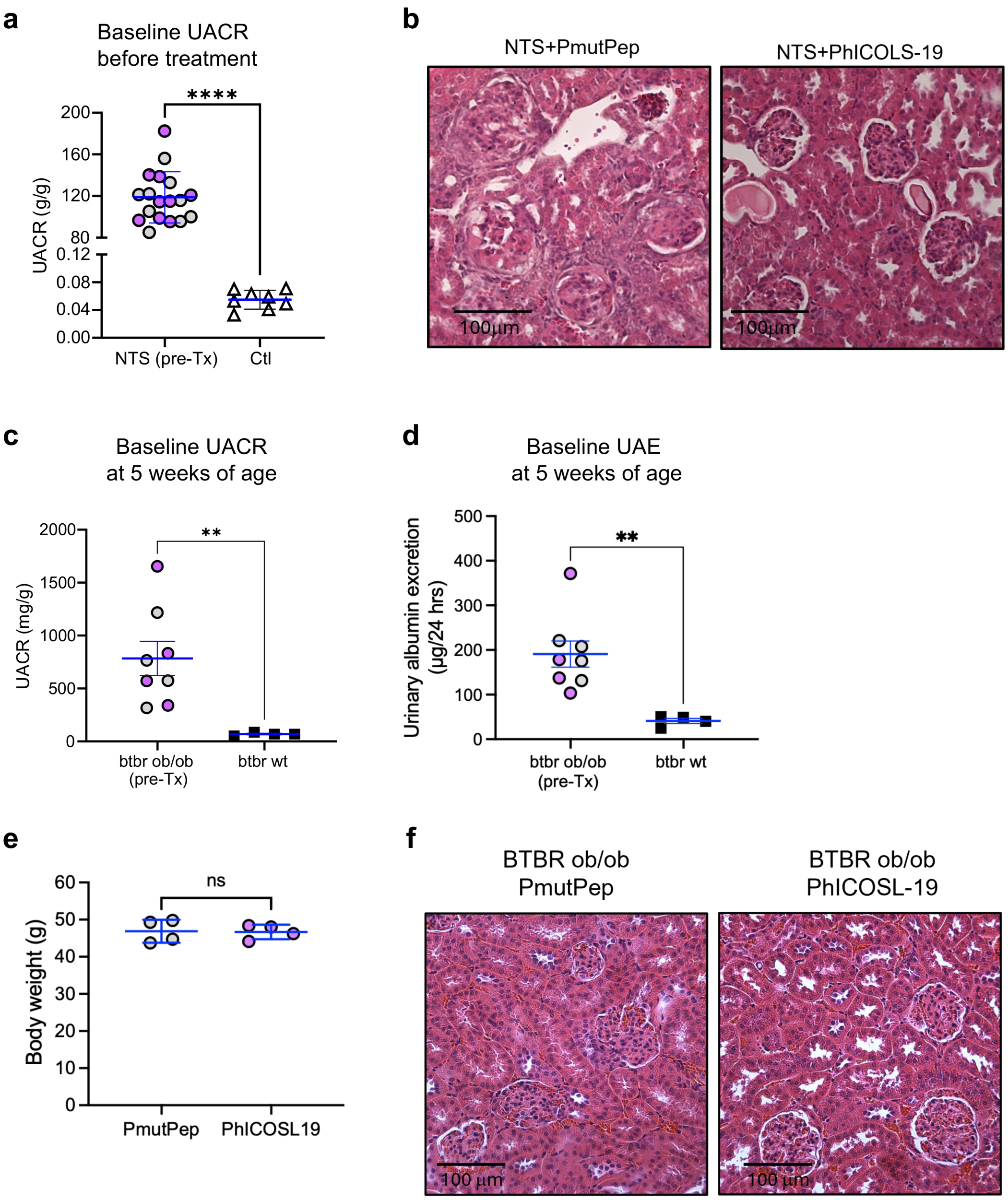

Figure S7. PhICOSL-19 treatment reduces glomerular injury in established proteinuric mice in two CKD models (active glomerulonephritis and diabetic nephropathy). (**a**) Baseline urinary albumin-to-creatinine ratio (UACR) in mice with NTS-induced glomerulonephritis before treatment initiation compared with untreated healthy controls. Purple circles indicate mice subsequently assigned to PhICOSL-19 treatment, gray circles indicate mice assigned to PmutPep control peptide treatment, and open triangles indicate healthy untreated controls without NTS challenge. (**b**) Representative H&E-stained kidney sections from NTS mice treated with PmutPep or PhICOSL-19. (**c**-**d**) Baseline proteinuria in 5-week-old BTBR ob/ob mice before treatment initiation compared with age-matched BTBR WT controls, assessed by (**c**) UACR and (**d**) 24-hour urinary albumin excretion (UAE). Purple circles indicate mice subsequently assigned to PhICOSL-19 treatment, gray circles indicate mice assigned to PmutPep control peptide treatment, and black squares indicate age-matched BTBR WT controls. (**e**) Body weight of BTBR ob/ob mice treated with PmutPep or PhICOSL-19. (**f**) Representative H&E-stained kidney sections from BTBR ob/ob mice treated with PmutPep or PhICOSL-19. Images are shown at 40x magnification; scale bars, 100 µm. Data are presented as mean ± SD and analyzed using unpaired two-tailed t-tests. $^{**}P \leq 0.01$, $^{****}P \leq 0.0001$; ns, not significant.

a

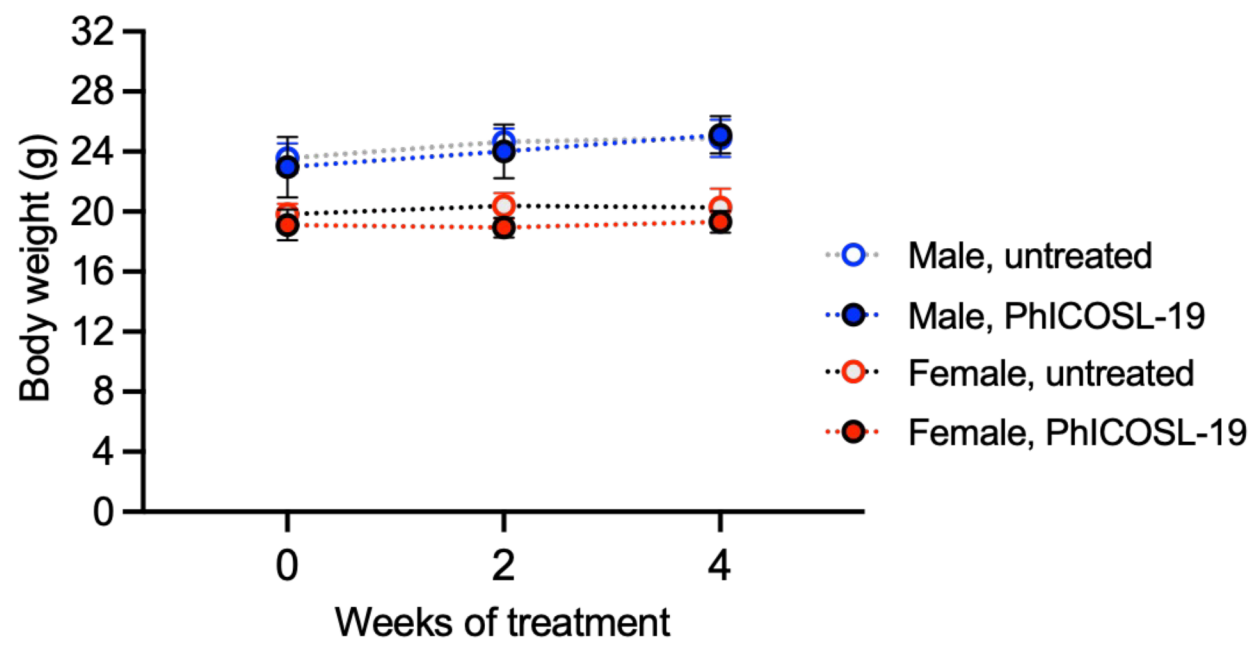

b

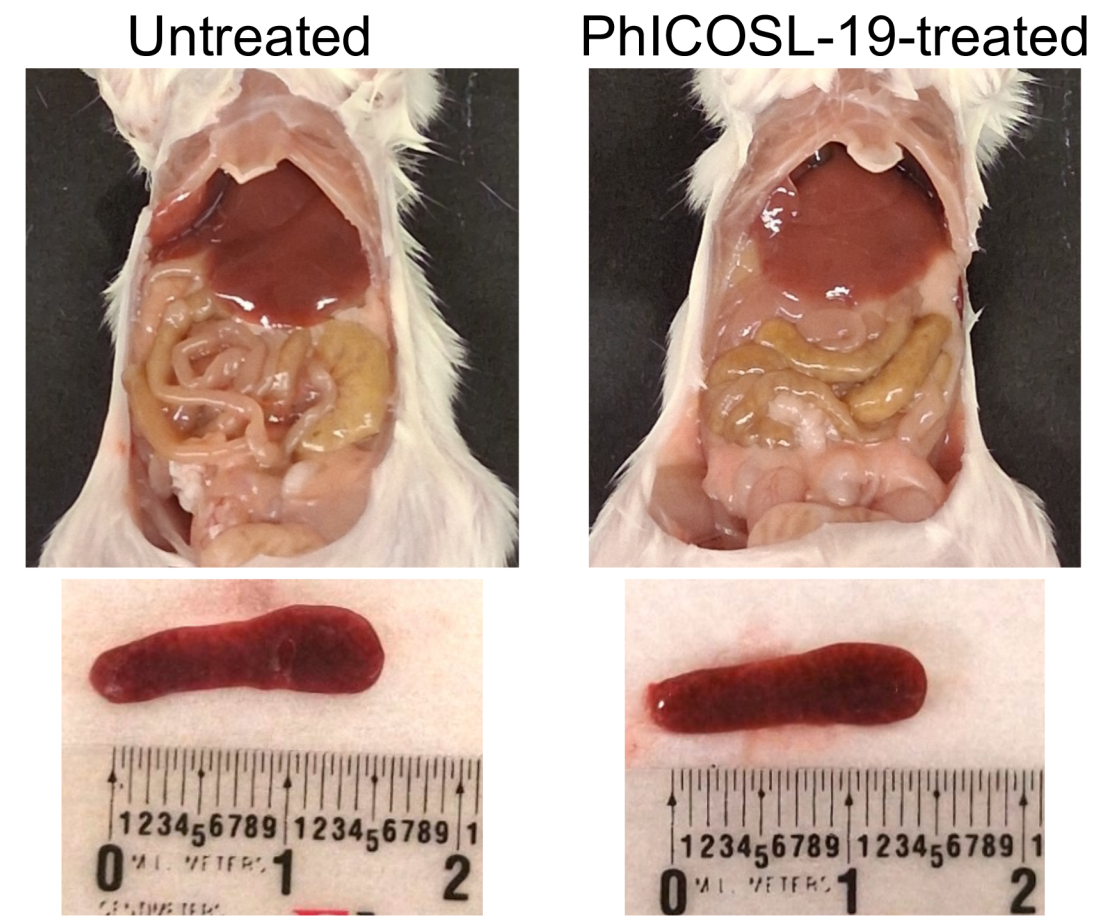

c

Spleen length and weight

Group	Sex	Spleen length (cm)	Spleen weight (g)
Untreated	F	1.6	0.1
Untreated	F	1.6	0.1
Untreated	F	1.6	0.1
Untreated	F	1.6	0.1
Untreated	F	1.6	0.1
Untreated	M	1.5	0.1
Untreated	M	1.6	0.1
Untreated	M	1.4	0.1
Untreated	M	1.5	0.1
Untreated	M	1.6	0.1
PhICOSL-19	F	1.6	0.1
PhICOSL-19	F	1.5	0.1
PhICOSL-19	F	1.6	0.1
PhICOSL-19	F	1.6	0.1
PhICOSL-19	F	1.6	0.1
PhICOSL-19	M	1.5	0.1
PhICOSL-19	M	1.5	0.1
PhICOSL-19	M	1.4	0.1
PhICOSL-19	M	1.3	0.1
PhICOSL-19	M	1.4	0.1

d

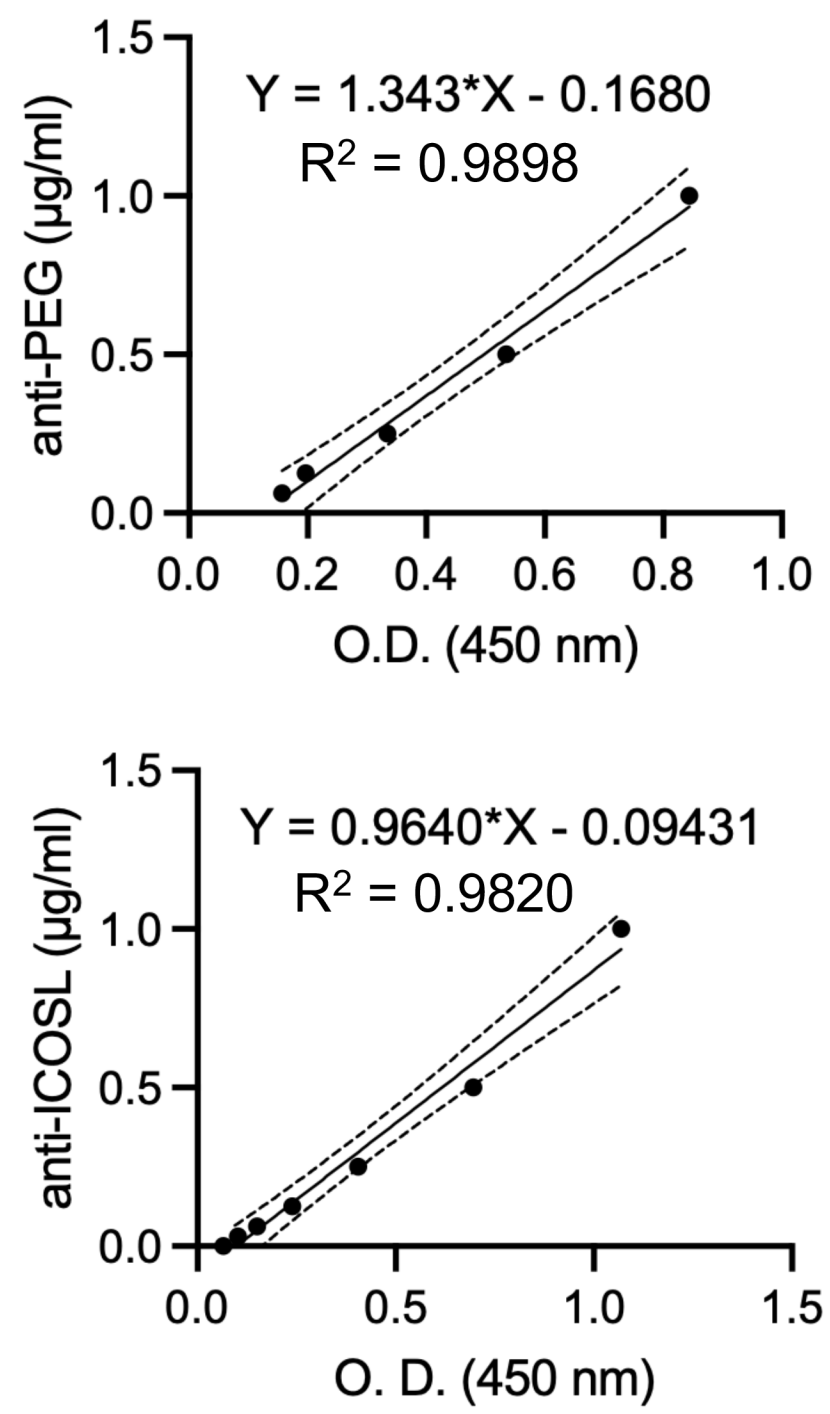

Figure S8. *In vivo* safety assessment of PhICOSL-19. (**a**) Body weight of male and female WT BALB/c mice receiving daily intravenous PhICOSL-19 (500 µg/kg) or left untreated, measured at weeks 0, 2, and 4. (**b**) Representative images from macroscopic examination of organs collected from untreated control and PhICOSL-19-treated mice at the end of the 4-week treatment period. (**c**) Spleen size and weight in untreated control and PhICOSL-19-treated mice. (**d**) Standard curves generated using mouse anti-PEG monoclonal IgG and rabbit anti-ICOSL polyclonal IgG as positive controls for the anti-drug antibody ELISA.

Materials and Methods

Table 1. Key Reagent or Resource

REAGENT OR RESOURCE	SOURCE	IDENTIFIER
**Antibodies**		
Mouse anti-human αvβ3 antibody	Millipore Sigma	MAB1976
Rabbit anti-human ICOSL antibody	GenScript	Custom-made and validated [28]
Normal rabbit IgG	Santa Cruz Biotechnology	sc-2027
Normal mouse IgG	Santa Cruz Biotechnology	sc-2025
Rabbit anti- FAK antibody	Cell Signaling	3285T
Rabbit anti-phospho-FAK (Tyr397) antibody	Cell Signaling	3283S
Rabbit anti-GAPDH antibody	abcam	ab9485
800CW Donkey anti-rabbit IgG	LicorBio	926-32213
680RD Donkey anti-rabbit IgG	LicorBio	926-68073
Anti-CD3/AF700	BD Boscience	557943
Anti-CD3/Pacific blue	Biolegend	100214
Anti-CD4/ FITC	Biolegend	100406
Anti-CD69/BV421	BD Bioscience	562884
Anti-CD69/PE Cy7	BD Bioscience	561930
Mouse monoclonal anti-PEG IgG	Millipore Sigma	MABS1966-25UG
HRP-conjugated goat anti-mouse IgG	Jackson ImmunoResearch	115-035-008
HRP-conjugated goat anti-rabbit IgG	Abcam	Ab205718
**Biological samples**		
De-identified human kidney biopsy samples, diabetic nephropathy	Kindly provided by Dr. David Cimbaluk (Dep. of Pathology, Rush University Medical Center)	
**Chemicals, peptides, and recombinant proteins**		
Full length human ICOSL (hICOSL) protein	Purified as previously described [28]; in-house	
hICOSL-19 peptide (RALMSPAGMLRGDFSLRLF)	GenScript	Custom-synthesized
Scramble peptide (SRALFMADMGRLLPSGRLF)	GenScript	Custom-synthesized
PhICOSL-19 ([PEG12]RALMSPAGMLRGDFSLRLF)	GenScript	Custom-synthesized
PmutPep ([PEG12]RALMSPAGMLAAAFSLRLF)	GenScript	Custom-synthesized
FITC-Ahx-hICOSL-19 peptide	GenScript	Custom-synthesized
cRGDfV	Enzo	BML-AM100-0001
$MnCl_2$	Millipore Sigma	M1787-1ML
Mouse vitronectin	Innovative Research	IMSVTNMULTI100UG
Human vitronectin	ACRO Biosystems	VIN-H5214
Bovine serum albumin	Thermo Fisher	BP9706100
RPMI 1640	Gibco	11875093
FBS	Denville Scientific	FB5001-H
Penicillin/Streptomycin	Gibco	15140122
Insulin-Transferrin-Selenium	Gibco	41400045

Collagen I	Corning	354236
Recombinant mouse interferon gamma	Cell Sciences	CR2041
Mesangial cell medium	ScienCell	4201
Endothelial cell medium	ScienCell	1001
Poly-L-lysine	Sigma Aldrich	P4707-50ML
Bovine plasma fibronectin	ScienCell	8248
Recombinant human IL-1 beta	Biolegend	579402
Recombinant human TNF alpha	R&D Systems	210-TA-020/CF
LPS	Invivogen	tlrl-eblps
Recombinant human integrin αvβ1 protein	R&D Systems	6579-AVB
Recombinant human integrin αvβ3 protein	R&D Systems	3050-AV-050
Recombinant human integrin αvβ6 protein	R&D Systems	3817-AV-050
Recombinant human integrin αvβ8 protein	R&D Systems	4135-AV-050
Recombinant human integrin α5β1 protein	R&D Systems	11538-A5
Recombinant mouse integrin αvβ3 protein	R&D Systems	7889-AV
CM5 sensor chip for SPR	Cytiva	BR100530
Nephrotoxic serum; sheep anti-rat GBM serum	Probetex	PTX-001S
Complete Freund's Adjuvant	Millipore Sigma	F5881
IgG from sheep serum	Millipore Sigma	I5131
AF488-conjugated Phalloidin	Invitrogen	A12379
Alarm blue	Invitrogen	DAL1025
Fixable Aqua Dead cell	Invitrogen	L34957
ELISA Buffer set	Thermo Fisher	CNB0011
**Critical commercial assays**		
PLA Duo link	Sigma Aldrich	DUO92102
RNeasy mini kit	Qiagen	74106
High-capacity cDNA reverse transcription kit	Applied Biosystems	4368814
Mouse albumin ELISA kit	Fortis Life Science	E99-134
Quantichome creatinine assay kit	BioAssay Systems	DICT-500
QuantiTect Reverse Transcription kit	Qiagen	205311
iTaq Universal SYBR Green Supermix	BioRad	1725120
PEGylated protein ELISA kit	Enzo	ADI-900-213
CellTiter-Glo	Promega	G7570
CyQuant LDH cytotoxicity assay	Invitrogen	C20300
**Experimental models: Cell lines**		
Human podocytes	Originally generated by Dr. Moin A. Saleem; kindly provided by Dr. Jochen Reiser	
Mouse podocytes	Originally generated by Dr. Peter Mundel; kindly provided by Dr. Jochen Reiser	
Human renal mesangial cells	ScienCell	4200
Human glomerular endothelial cells	ScienCell	4000

**Experimental models: Organisms/strains**		
Icosl KO mice (backcrossed to BALB/c mice as previously described) [56]	Kindly provided by Dr. Alison Finnegan (Dep. of Immunology, Rush University Medical Center)	
BALB/c WT mice	Jackson laboratory	stock # 000651
BTRB *ob/ob* mice; BTBR.Cg-*Lep*^{ob}/WiscJ,	Jackson laboratory	stock # 004824
BTBR/WT	Jackson laboratory	Stock #002282
**Oligonucleotides**		
TaqMan gene probe for mouse *Icosl*	ThermoFisher	Mm00497238_m1
TaqMan gene probe for human *ICOSLG*	ThermoFisher	Hs00323621_m1
TaqMan gene probe for mouse *Gapdh*	ThermoFisher	Mm99999915_g1
TaqMan gene probe for human *GAPDH*	ThermoFisher	Hs02758991_g1
**Public databases**		
Human ICOSL amino acid sequence	UniProt	O75144
Mouse ICOSL amino acid sequence	UniProt	Q9JHJ8
Glomerular ICOSLG expression	Nephroseq	Ju CKD Glom Dataset

Cell culture

Conditionally immortalized mouse and human podocytes were cultured and differentiated as previously described [39, 40, 70]. Podocytes were maintained at 33°C for proliferation and differentiated at 37°C for 10-14 days. Unless otherwise indicated, all experiments were performed using differentiated podocytes. Human podocytes were cultured in RPMI-1640 medium supplemented with 10% FBS, 1X insulin-transferrin-selenium (ITS; Gibco, 41400045), and 1% penicillin/streptomycin. Mouse podocytes were cultured on culture flasks coated with 0.1 mg/mL collagen I (Corning, 354236) in RPMI-1640 medium containing 10% FBS, 1% penicillin/streptomycin, and recombinant mouse interferon-γ (10 U/mL; Cell Sciences, CR2041) at 33°C, and differentiated at 37°C in medium containing 2% FBS and 1% penicillin/streptomycin without interferon-γ. Human renal mesangial cells (ScienCell, 4200) were cultured in mesangial cell medium (ScienCell, 4201) in 2 μg/cm^2 poly-L-lysine (Sigma, P4707-50ML)-coated culture flasks at 37°C. Human renal glomerular endothelial cells (ScienCell, 4000) were cultured in endothelial cell medium (ScienCell, 1001) in 2 μg/cm^2 fibronectin (ScienCell, 8248)-coated culture flasks at 37°C. Jurkat T cells were maintained in RPMI-1640 medium supplemented with 10% FBS and 1% penicillin/streptomycin at 37°C in a humidified atmosphere containing 5% CO_2.

Surface plasmon resonance (SPR)

All assays were performed on a Biacore T200 instrument (GE Healthcare) as previously described[28]. Before immobilization, sensor surface was activated by 1-Ethyl-3-(3-dimethylaminopropyl) carbodiimide/ N-hydroxysuccinimide) (EDC/NHS). Full length hICOSL protein, purified as previously described[28], custom-synthesized hICOSL-19, PhICOSL-19, and scrambled control peptides (GenScript) were immobilized on flow channels of a CM5 sensor chip using a standard amine-coupling method. After immobilization, the unoccupied chip surface was blocked by ethanolamine. The candidate analytes, human or mouse integrins, were applied through the channels at a 20-25 μL/minute flow rate at 25°C at several concentrations (0 to 160 nM). The binding experiments were performed in HEPES binding buffer (10 mM HEPES, 150 mM NaCl, 0.05% n-Octyl-β-D-glucopyranoside, pH 7.1) supplemented with 2 mM $MnCl_2$ as a source of Mn^{2+} for integrin activation. For interaction inhibition assays, human or mouse αvβ3 integrins were preincubated with cRGDfV (Enzo, BML-AM100-0001) at 30 μM and then perfused through the channels. Data were double referenced with zero concentration analyte resonance unit (RU) and blank RU values on flow channel 1. Biacore T200 evaluation software 2.0.3 was used to analyze the sensorgrams and measure RU during the equilibration phase at each concentration for steady-state affinity fittings. Kinetic fittings and affinity fittings were determined by 1-to-1 Langmuir binding model embedded within the Biacore T200 evaluation software 2.0.3. Rate constants (ka and kd) were determined by kinetic fitting or

affinity fitting of the sensorgrams using 1-to-1 Langmuir binding equation, and K_D (equilibrium disassociation constant) values were calculated by kd/ka.

***In situ* proximity ligation assay (PLA)**
PLA was conducted utilizing the Duolink in Situ Orange Starter Kit for Mouse/Rabbit antibodies (Sigma Aldrich, DUO92102) according to the manufacturer's instructions. The assay was conducted in $MnCl_2$-treated differentiated human podocytes grown on coverslips and in de-identified frozen kidney biopsy specimens from individuals with early stage of diabetic nephropathy (n = 7). Kidney biopsy specimens were provided by Dr. David Cimbaluk, Department of Pathology, Rush University Medical Center, Chicago, IL, USA. The retrospective use of these de-identified human kidney biopsy specimens was approved by the Rush University Medical Center Institutional Review Board (IRB no. 14051401). Podocytes (on coverslips) and kidney biopsies sections were rinsed with ice-cold PBS and fixed with 4% PFA for 10 minutes at room temperature. Subsequently, permeabilization was achieved with 0.1% Triton X-100 for 5 minutes. After two 5-minute washes with PBS, the tissues underwent blocking for 1 hour with agitation in Duolink PLA blocking solution. Following the blocking step, slides were incubated overnight at 4°C with a combination of either rabbit anti-ICOSL (10 µg/mL; custom GenScript)[28] and mouse anti-β3 (10 µg/mL; Millipore Sigma, MAB 1976). Normal rabbit IgG (10 µg/mL; Santa Cruz Biotechnology, sc-2027) and normal mouse IgG (10 µg/mL; Santa Cruz Biotechnology, sc-2025) were used as negative controls. Samples were then incubated with complementary oligonucleotide-conjugated PLA probes, anti-rabbit PLUS (DUO92002) and anti-mouse MINUS (DUO92004), for 1 hour at 37°C, followed by ligation for 30 minutes, amplification for 100 minutes, and washing with Buffers A and B according to the manufacturer’s protocol. All incubations were performed in a humidified chamber. Samples were counterstained with DAPI, and confocal images were acquired using an LSM 700 laser-scanning confocal microscope with ZEN software (Zeiss).

ICOSL expression in glomerular cells under cellular stress
To evaluate ICOSL expression in response to cellular stress, differentiated human podocytes, human glomerular endothelial cells (GEnC), and human mesangial cells (MC) were exposed to immunological stimuli, whereas differentiated human and mouse podocytes were subjected to mechanical stretch, followed by analysis of *ICOSLG*/*Icosl* mRNA expression. For immunological stress induction, human podocytes, GEnC, and MC were stimulated with IL-1β (BioLegend, 579402), TNFα (R&D Systems, 210-TA-020/CF), or LPS (InvivoGen, tlrl-eblps) at 20 ng/mL, 100 ng/mL, or 50 µg/mL, respectively, for 3 hours. Total RNA was isolated using a RNeasy Mini Kit (Qiagen, 74106), and cDNAs were synthesized using the High-Capacity cDNA Reverse Transcription Kit (Applied Biosystems, 4368814). qPCR was performed in duplicate using a CFX96 Real-Time System (Bio-Rad) and TaqMan probes for mouse *Icosl* (Mm00497238_m1), human *ICOSLG* (Hs00323621_m1), mouse *Gapdh* (Mm99999915_g1), and human *GAPDH* (Hs02758991_g1). Gene expression was normalized to GAPDH and expressed as fold change relative to untreated controls using the $2^{-\Delta\Delta Ct}$ method. Mechanical stretch experiments were performed as previously described[41]. Briefly, differentiated mouse or human podocytes were seeded on collagen IV pre-coated flexible silicone membranes of a six-well plate (Bioflex, Flexcell® International Corporation, Burlington, NC, USA). After three days, the six-well plate was mounted on a manifold connected to a custom-built stretch apparatus (NIPOKA GmbH, Greifswald, Germany), which then induced cyclic pressure variations resulting in upward and downward motion of the silicone membranes. Pressure amplitude was chosen to give a maximum upward deflection of the membrane center of 6 mm being equal to an increase in membrane area by 11%, or 5% mean linear cell strain. The human podocytes were additionally stretched with a higher amplitude (8.5 mm). The cycle frequency was adjusted to 0.5 Hz. This frequency was chosen because autoregulation of renal blood flow can damp pressure fluctuations below 0.1 Hz, whereas faster pressure fluctuations (> 0.1 Hz) are fully transmitted to glomerular capillaries. Following mechanical stimulation, cells were collected in Tri-Reagent (Sigma-Aldrich), and total RNA was isolated according to the manufacturer’s instructions. cDNA was synthesized using the QuantiTect Reverse Transcription Kit (Qiagen, Hilden, Germany), and qPCR was performed using a QuantStudio™ 5 Real-Time PCR System (Thermo Fisher Scientific) with iTaq Universal SYBR Green Supermix (Bio-Rad). Relative mRNA expression was normalized to GAPDH and calculated using the efficiency-corrected Pfaffl method from at least three biological replicates. Primer sequences were as follows: mouse *Icosl,* forward 5’CATTAAGGCTCCCTTGGACA3’ and reverse 5’CTTTCTGCCTGGCTAATGCT3’; human *ICOSLG*, forward 5’CAGGACGAGCAGAAGTTTCA3’ and reverse 5’GAAGTTTGCTGCCACATGC3’; mouse *Gapdh*, forward

5'TGAAGCAGGCATCTGAGGG3' and reverse 5'CGAAGGTGGAAGAGTGGGAG3'; human *GAPDH*, forward 5'GTTCGACAGTCAGCCGCATC3' and reverse 5'AGTTAAAAGCAGCCCTGGTGA3'.

Podocyte adhesion assay

For direct adhesion assays, hICOSL-19 (100 µg/mL), PhICOSL-19 (100 µg/mL), hICOSL (20 µg/mL), vitronectin (1 µg/mL), or BSA (20 µg/mL) were used to coat the corresponding wells of a 384-well plate overnight at 4°C and blocked with BSA 1% during 1 hour at 37°C. The differentiated immortalized human podocytes were pre-treated as indicated in each figure legend with cRGDfV (10 µg/mL), anti-β3 antibody (10 µg/mL Millipore Sigma, MAB 1976), normal mouse IgG (10 µg/mL), hICOSL-19 (100 µg/mL, PhICOSL-19 (100 µg/mL) or PBS for 15 minutes prior to treatment with $MnCl_2$ (250 µM). After treatment, cells were seeded into the precoated 384-well plate (5000 cells/well) and were then incubated at 37°C for 2 hours. After incubation, the unadhered cells were washed off the plate, and the adhered cells were fixed with 4% PFA for 30 minutes followed by DAPI staining to visualize cell nuclei. The DAPI stained cells in each well were imaged using a Nikon eclipse Ti2 microscope. The total adhered cell numbers in each well were counted using Nikon's NIS elements AR software. For adhesion assays using mouse podocytes, the same experimental procedure was used with a few adjustments: hICOSL-19 (40 µg/mL), scramble peptide (40 µg/mL), PhICOSL-19 (40 µg/mL), hICOSL (20 µg/mL), mouse vitronectin (20 µg/mL) or BSA (20 µg/mL) were used for coating a 384-well plate for overnight at 4°C. The differentiated immortalized mouse podocytes were pre-incubated with hICOSL-peptide (20 µg/mL), cRGDfV (10 µg/mL), or PBS prior to the treatment with $MnCl_2$ (250 µM). After loading cells onto the precoated plates, cells were incubated for 2 hours at 37°C. For the competitive adhesion assay, a similar procedure was carried out, coating all the wells with the vitronectin (1 µg/mL), and pre-treating the cells with the corresponding peptides (cRGDfV, hICOSL-19, PhICOSL-19), and controls (PBS as vehicle). Measurement was interpreted as adhesion reduction to the cognate ligand vitronectin. For all measurements, relative adhesion was obtained as the average of the maximum adhesion (i.e. adhesion to vitronectin) normalized to 100%.

Western blot

Human podocytes were pretreated with vehicle (1% BSA), hICOSL-19 (100 µg/mL), PhICOSL-19 (100 µg/mL), or cRGDfV (10 µg/mL) for 20 minutes and then plated onto vitronectin- coated plates (10 µg/mL; Innovative Research, IMSVTNMULTI100UG) in the presence of $MnCl_2$ (250 µM) for 45 minutes. Cell lysates were subsequently collected for Western blot analysis. Total FAK and phosphorylated FAK (Tyr397) were detected using rabbit anti-FAK (Cell signaling, 3285T) and anti-phospho-FAK (Tyr397) (Cell signaling, 3283S) antibodies, respectively, at 1:1,000 dilution. IRDye 800CW and IRDye 680RD secondary antibodies (LicorBio, 926-32213, 926-68073) were used at 1:15,000 dilution, and signals were visualized using Li-Cor Odyssey system. All images were converted to gray scale, and band intensities were quantified using Image J, and the phospho-FAK/FAK ratio was normalized to the vehicle-treated control.

Animals

Icosl-KO mice backcrossed onto the BALB/c background, as previously described [71], were obtained from Dr. Alison Finnegan (Rush University Medical Center, Chicago, Illinois, USA). BALB/c (stock# 000651), BTBR ob/ob (BTBR.Cg-*Lep^ob*/WiscJ, stock# 004824), and BTBR/WT mice (BTBR T+ tf/J, stock # 002282) were obtained from the Jackson Laboratory. All animal experiments were carried out according to the NIH Guide for Care and Use of Experimental Animals and approved by Rush University's Institutional Animal Care and Use Committee (IACUC protocol #23-066). Mice were housed in the animal facility at Rush University Medical Center under specific pathogen-free (SPF) conditions, with a 12-h light/12-h dark cycle and *ad libitum* access to standard chow and water. Animal rooms were maintained at approximately 72 °F and 45% relative humidity. Sample sizes were chosen based on previous experience and publications in our laboratory. Animals within each experiment were age- and sex-matched, and the number of animals used in each experiment is provided in the corresponding figure legends. Animals were euthanized by carbon dioxide inhalation or isoflurane anesthesia followed by cervical dislocation, according to the approved IACUC protocol, and tissues were subsequently collected for analysis. For all *in vivo* efficacy studies, PhICOSL-19 was administered at 500 µg/kg body weight. This dose was selected for initial proof-of-concept studies based on our previous efficacy studies using full-length ICOSL[28] and published dosing ranges of integrin-targeting peptides in mice[72, 73].

In vivo evaluation of PhICOSL-19 therapeutic efficacy

PhICOSL-19 therapeutic efficacy was evaluated in three complementary models of proteinuric kidney disease. For the passive anti-GBM nephritis model, acute glomerulonephritis was induced in 15-week-old female Icosl-KO mice by intravenous (IV) administration of nephrotoxic serum (NTS; sheep anti-rat GBM serum; Probetex, PTX-001S) at 1 mL/kg body weight. Mice were then treated with PhICOSL-19 (IV, 500 µg/kg body weight) or saline at 1 hour and 24 hours after NTS challenge, and proteinuria was evaluated daily for 2 days following treatment initiation. For the chronic active NTS nephritis model, 10-week-old female BALB/c WT mice were pre-immunized with sheep IgG (Millipore Sigma, 200 µg/mouse) emulsified in complete Freund's adjuvant (CFA) at a 1:1 ratio. Four days later, mice received NTS (sheep anti-rat GBM serum; 100 µL/mouse) to induce a secondary immune response. Before treatment initiation, urinary albumin-to-creatinine ratio (UACR) was measured to confirm the development of proteinuria, after which mice were randomly assigned to treatment groups. Starting 2 days after NTS injection, mice received daily treatment of PhICOSL-19 or PEGylated mutant peptide (IV, 500 µg/kg body weight) for 5 consecutive days. Proteinuria and renal histopathological changes were assessed one day after the final treatment. For the metabolic kidney disease model, 5-week-old male BTBR ob/ob mice were used as a model of diabetic kidney disease. Prior to treatment initiation, UACR was performed to confirm proteinuria development, next the mice were randomly divided into two groups. Mice then received daily treatment of PhICOSL-19 or PEGylated mutant control peptide (IV, 500 µg/kg body weight) for 6 consecutive weeks. One day after the final treatment, proteinuria was confirmed by measurement of UACR and urinary albumin excretion (UAE) using metabolic cage urine collections, and kidneys were collected for histopathological evaluation.

Urinary albumin-to-creatinine ratio (UACR) and urinary albumin excretion (UAE) measurements

Mouse urine samples were collected to assess proteinuria. In the immune-mediated kidney injury models (passive and active NTS), spot urine samples were collected, whereas in the metabolically mediated kidney injury model, 24-hour urine samples were collected using metabolic cages. Urinary albumin and creatinine concentrations were measured using a mouse albumin ELISA kit (Fortis Life Sciences) and a creatinine assay kit (BioAssay Systems), respectively, according to the manufacturers' protocols. UACR was calculated as the ratio of urinary albumin to urinary creatinine. For 24-hour urine collections, urinary albumin excretion (UAE) was calculated from the urinary albumin concentration and total urine volume collected over 24 hours. UACR and UAE were used as measures of proteinuria.

Histology

Formalin-fixed kidneys, paraffin-embedded kidney tissues were prepared for hematoxylin and eosin (H&E) and periodic acid-Schiff (PAS). Samples were sectioned to a thickness of 5 µm. Glomerular injury score was evaluated in 20-50 glomeruli per sample of the H&E staining using semi-quantitative scoring scale 0 to 4, where 0 = absent/normal, 1 = minimal (<10%), 2 = mild (10-25%), 3 = moderate (26-50%), and 4 = severe (>50%) based on hypercellularity, matrix increase, sclerosis, and crescent formation. Histological evaluations were performed by Dr. David Cimbaluk, Renal Pathology Laboratory director at Rush University Medical Center.

Electron microscopy

Morphological analysis of samples using transmission electron microscopy (TEM) was performed as previously described[28]. Briefly, samples were fixed in 4% paraformaldehyde (PFA) overnight at 4°C then post-fixed in 1% osmium tetroxide (OsO_4) for 1 hour on ice. Samples were rinsed in 1% cacodylate buffer and dehydrated in a PBS/ethanol series of washes to 100% ethanol. Samples were then transferred to 100% propylene oxide and infiltrated with Epon resin (Embed 812, Electron Microscopy Sciences) following the manufacturer's instructions. Hardened resin blocks containing samples were sectioned on a Leica EM UC7 ultramicrotome to a thickness of 100nm. EM grids (formvar coated nickel slot grids, Electron Microscopy Sciences) with ultrathin sections were stained using 2% uranyl acetate and 0.5% lead citrate then imaged on a Zeiss Sigma HD VP electron microscope. Three mice from both the PhICOSL-19 treated group and the PEGylated mutant peptide group were sampled along with 2 healthy (naïve) control mice. Three glomeruli per mice were analyzed, a total of 5-9 images per glomerulus were taken for the statistical analysis.

In vivo safety and immunogenicity

Eight- to nine-week-old WT BALB/c mice (female, n = 5; male, n = 5) received PhICOSL-19 peptide (500 µg/kg) by daily tail-vein injection for 4 weeks, while age-matched untreated WT BALB/c mice (female, n = 5; male, n = 5) served as controls. Body weight was measured before treatment and at 2 and 4 weeks after treatment initiation. At the end of treatment, serum was collected for anti-drug antibody (ADA) assessment, and spleens were harvested for analysis of T-cell activation. Splenocytes were isolated by mechanical dissociation followed by red blood cell lysis. Cells were stained with Fixable Aqua Dead Cell Stain (Invitrogen, L34957), anti-CD45 (APC Cy7, Biolegend, 103116), anti-CD3 (Pacific Blue, Biolegend, 100214), and anti-CD69 (PE Cy7, BD Bioscience, 561930). T cells were identified as live CD45+CD3+ cells and activated T cells were defined as CD69+ within the gated CD3+ T-cell population. Data were analyzed using FlowJo v10.1. For ADA assessment, an indirect ELISA was developed to detect serum antibodies reactive to PhICOSL-19. Ninety-six-well plates were coated with PhICOSL-19 peptide (50 µg/ml, 100 µl/well) overnight at 4°C. Serum samples were diluted 1:2 in assay buffer (Thermo Fisher ELISA buffer set, CNB0011). Mouse anti-PEG monoclonal IgG (Millipore Sigma, MABS1966-25UG) and rabbit anti-ICOSL polyclonal IgG (GeneScript, custom) were included as positive controls and used to generate standard curves over a concentration range of 0.0313-1 µg/ml. HRP-conjugated goat anti-mouse IgG (Jackson ImmunoResearch, 115-035-008; 1:10000 dilution) was used for serum samples and the anti-PEG standard curve, while HRP-conjugated goat anti-rabbit IgG (Abcam, ab205718; 1:10000 dilution) was used for the anti-ICOSL standard curve. TMB substrate and sulfuric acid stop solution were applied according to the manufacturer's instructions, and absorbance was measured at 450 nm. Serum antibody reactivity against PhICOSL-19 was evaluated by comparing absorbance values between PhICOSL-19-treated and untreated control mice.

Pharmacokinetic (PK) analysis

Blood samples (~30 µL) were obtained from the retro-orbital plexus of 12-week-old male Icosl-KO mice at 0.05, 0.5, 1-, 3-, 6-, and 24-hours post-administration of the peptide and serum was isolated by centrifugation. Pharmacokinetic analysis of FITC-hICOSL-19 (n=3) was performed after diluting samples 4-fold in PBS and then measuring fluorescence on a Synergy LX multimode plate reader (BioTek) using 485 nm excitation and 520 nm emission wavelengths. Pharmacokinetic analysis of PhICOSL-19 was performed using a PEGylated protein ELISA kit (Enzo, ADI-900-213) after serum samples were diluted 8-fold in PBS and measuring absorbance at 450 nm on a Synergy LX multimode plate reader (BioTek). Data at each time point were subjected to analysis for fitting to the equation representing a two-phase decay using Prism version 9.4 (GraphPad).

In vitro safety assays

Differentiated human podocytes were seeded in 96 well plates ($1x10^4$/well) and treated with vehicle (PBS), adriamycin as a positive control of damage (1 µg/ml), and PhICOSL-19 peptide (2, 10, and 20 µg/ml). Treatments were added every 24 h for 96 h. Cells were then assessed for cytoskeleton alterations, cell viability, and cell cytotoxicity. For cytoskeleton evaluation, cells were stained with DAPI and AF488-conjugated Phalloidin (Invitrogen, A12379) for F-actin cytoskeleton evaluation. Briefly, after cells were fixed with 4% PFA and permeabilized with PBS + Triton 0.1%. After permeabilization cells were incubated with AF488-conjugated Phalloidin (Dilution 1:50 from stock solution) and DAPI for nuclei counterstaining. Images were acquired with an EVOS M5000 microscope. Cell viability was assessed using CellTiter-Glo (Promega, G7570) and AlamarBlue (Invitrogen, DAL1025) according to manufacturers' instructions. These assays measure ATP content and NAD(P)H dehydrogenase activity, respectively, as indicators of metabolically active cells. CellTiter-Glo results were obtained as luminescence (RLU), while AlamarBlue fluorescence was measured using excitation/emission wavelengths of 540-570/580-610 nm. Cell cytotoxicity was assessed using CyQuant LDH cytotoxicity assay (Invitrogen, C20300) according to manufacturer's instructions. Extracellular lactate dehydrogenase (LDH) activity was measured at 490 nm with background absorbance at 680 nm subtracted from each well. Percentage cytotoxicity was calculated as *100 x [(treatment condition - spontaneous LDH)/(Maximum LDH - spontaneous LDH),* where spontaneous LDH release was determined from control cells treated with 10 µL ultrapure water and maximum LDH release was determined from control cells treated with 10 µL lysis buffer, each performed in triplicate.

In vitro T-cell activation assay

Jurkat T cells and peripheral blood mononuclear cells (PBMC) from healthy donors were used to evaluate whether PhICOSL-19 incudes T-cell activation. Cells ($3x10^5$ cells/well) were seeded in 48-well plates in RPMI medium supplemented with 10% FBS and treated with PhICOSL-19 at 2, 10, or 20 µg/mL. Phorbol 12-myristate 13-acetate (PMA; 100 ng/mL) plus ionomycin (1 µg/mL) was used as a positive control, and vehicle (PBS) served as a negative control. Following stimulation, cells were stained with Fixable Aqua Dead Cell Stain (Invitrogen, L34957), anti-CD3 (AF700; BD Biosciences, 557943), and anti-CD69 (BV421; BD Biosciences, 562884). CD69 expression was assessed as an early marker of T-cell activation. Flow cytometry data were analyzed using FlowJo v10.1. For Jurkat cells, CD69 expression was quantified among live cells, whereas for PBMCs, CD69 expression was quantified within the live $CD3^+$ T-cell population.

Nephroseq

All patient's data were obtained from the public database Nephroseq (University of Michigan)[44]. Ju CKD Glom Dataset was used for graph presented in Fig. S4d. ICOSLG, CKD, and glomerulus were used as key words. JuCKD Glom Data[44, 45] set contains the gene expression profiling of 199 micro-dissected glomerular samples from CKD patients and living donors analyzed on Affymetrix Human U133 Plus 2.0 and Affymetrix Human U133A (altCDF v10) platforms.

Statistical analysis

Statistical analysis was performed utilizing Prism 10.2.1 software unless stated otherwise (GraphPad). Significance was determined with a threshold of $p \leq 0.05$, denoted as follows: *p ≤0.05, **p ≤ 0.01, ***p ≤ 0.001, ****p ≤ 0.0001, and referenced accordingly in the figure legend. T-tests were employed to compare differences between two groups. Differences among multiple groups were assessed using one-way ordinary or two-way ANOVA according to each experiment. Data are presented as means ± standard deviation (SD).
